# Endohedral Coordination of Bulky Substrates in Metalloenzyme‐Like Organometallic Nanotubes

**DOI:** 10.1002/chem.202500775

**Published:** 2025-05-03

**Authors:** Thomas Pickl, Patrick Mollik, Markus R. Anneser, Florian Sixt, Korbinian Geißer, Oksana Storcheva, Dominik P. Halter, Alexander Pöthig

**Affiliations:** ^1^ Department of Chemistry Catalysis Research Center (CRC) & TUM School of Natural Sciences Technical University of Munich Ernst‐Otto‐Fischer Str. 1 85747 Garching Germany; ^2^ Department of Biochemical and Chemical Engineering Research Group Applied Electrochemistry & Catalysis (ELCAT) Faculty of Applied Engineering University of Antwerp Universiteitsplein 1 Antwerp 2610 Belgium

**Keywords:** cavitands, metalloenzymes, nanotubes, organometallic, pillarplex

## Abstract

Artificial receptors inspired by metalloenzymes share three key properties: a structurally flexible cavity, substrate binding *via* metal‐ligand coordination, and metal‐based redox activity. Herein, we report an organometallic nanotube with such features based on our supramolecular pillarplex platform, incorporating eight Cu^I^ centers in its cavitand walls. The structurally adaptable cavity of this receptor enables the endohedral coordination of tetrahydrofuran (THF) as a hydrophilic model substrate with remarkable binding affinity despite a steric mismatch between the host and guest. Evidence from SC‐XRD, ^1^H NMR titration in aqueous solution, and DFT modelling confirms that metal‐ligand coordination governs substrate binding. Electrochemical analysis of a derived rotaxane reveals metal‐centered redox activity.

## Graphical abstract

An organometallic nanotube with eight Cu^I^ ions mimics metalloenzyme behavior, featuring a flexible pore, metal‐ligand substrate binding, and redox‐active metals. Insertion of THF as a model substrate is driven by coordination, as confirmed by SC‐XRD, NMR, and DFT. Electrochemical studies of a derived rotaxane reveal metal‐centered redox activity.

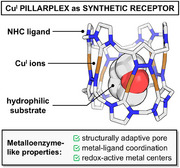

## 1 Introduction

**Figure 1:**
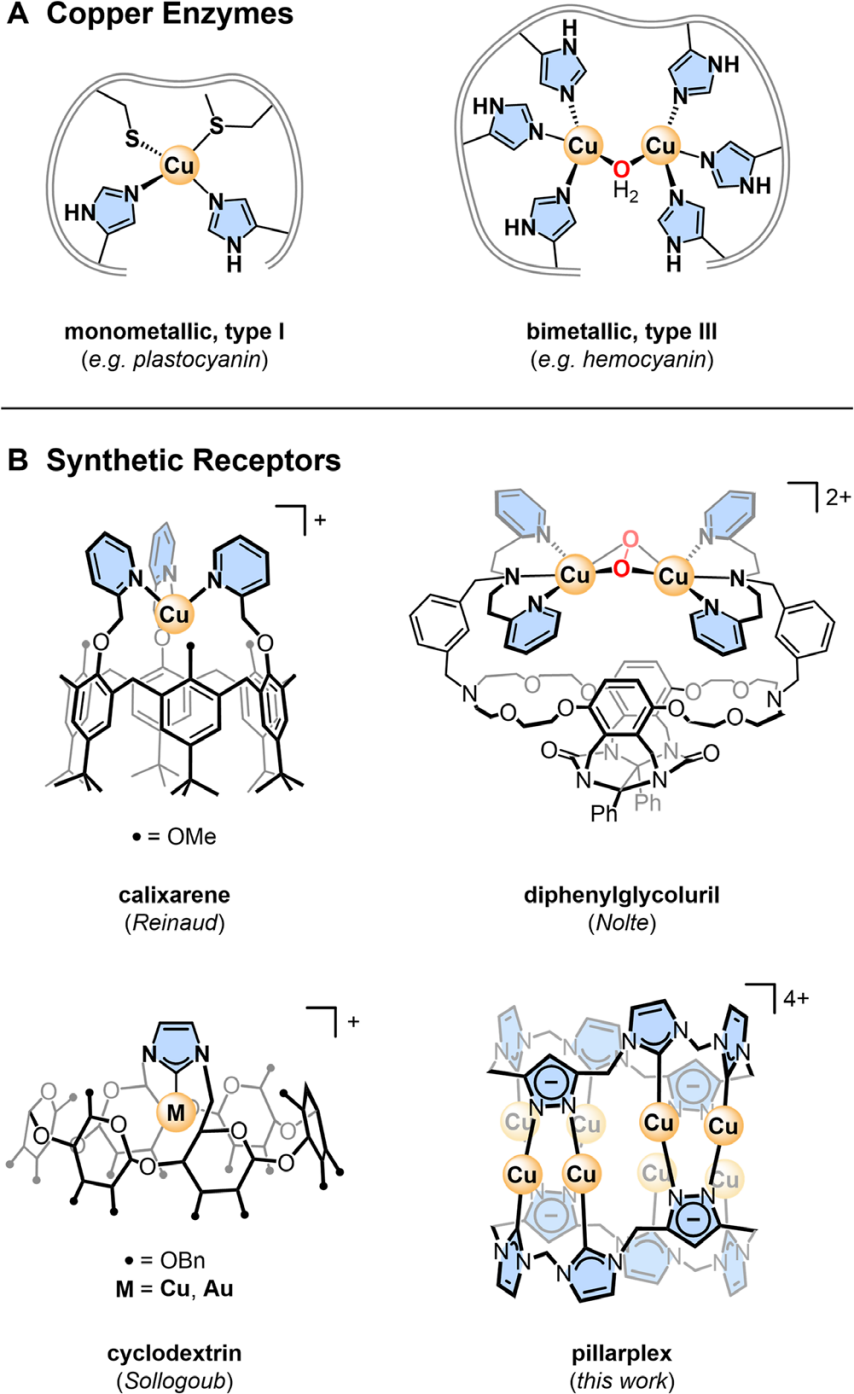
(**A)** Active sites of representative mono‐ and bimetallic copper enzymes; (**B)** Examples of biomimetic metal complexes derived from typical cavitand platforms.

Metalloenzymes are nanoscale “reactors” found in all living organisms, catalyzing a broad range of chemical transformations.^[^ ^1^ ^]^ Central to their function, these proteins incorporate metal ions within the cavity of their active sites. Conformational flexibility of this confined space is critical for efficient substrate binding, intermediate stabilization, and product release.^[^ ^2^, ^3^, ^4^ ^]^ In this context, type‐III copper enzymes stand out for their remarkable structural adaptability.^[5,6]^ Their flexible active sites contain two histidine‐bound copper ions (Figure 1A),^[^ ^7^ ^]^ whose distance can be dynamically adjusted by the enzyme (2.8 – 4.6 Å) based on substrate availability (e.g., O_2_).^[^ ^8^ ^]^ This underpins the function of polyphenol oxidases and hemocyanin, an oxygen transport protein found in certain arthropods.^[^ ^9^ ^]^

Synthetic receptors have been designed to mimic the unrivalled shape‐ and size‐selective guest binding and reactivity of metalloenzymes,^[^ ^10^ ^]^ including (type‐III) copper proteins.^[^ ^11^, ^12^, ^13^ ^]^ A simple approach toward enzyme mimics is to anchor a metal center to the rim of a pre‐formed cavitand, creating a well‐defined pocket for substrate binding (Figure 1B).^[^ ^14^ ^]^ Often, the metals are used to cap one pore opening, resembling the enzymatic reactive site, while the cavitand models an enzymatic substrate‐access channel.^[^ ^13^, ^15^, ^16^, ^17^, ^18^ ^]^ The pore dimensions and properties can be fine‐tuned by selecting specific cavitand platforms, such as cyclodextrins,^[^ ^19^, ^20^, ^21^, ^22^ ^]^ calixarenes,^[^ ^23^, ^24^, ^25^ ^]^ resorcinarenes,^[^ ^14^, ^15^, ^16^, ^17^, ^18^, ^19^, ^20^, ^21^, ^22^, ^23^, ^24^, ^25^, ^26^ ^]^ or diphenylglycolurils.^[^ ^27^, ^28^ ^]^

Herein, we introduce a tubular cavitand with internal coordination sites for substrate binding, reminiscent of multinuclear copper enzymes. Based on our pillarplex platform,^[^ ^29^ ^]^ the organometallic nanotube **[Cu_8_L_2_](OTf)_4_** features an uncapped pore that is accessible from both sides. In contrast to the aforementioned cavitands, our system integrates metal ions directly in the cavitand walls as the tetra‐cationic molecule comprises a ring of eight Cu^I^ ions sandwiched between two organic ligands (Figure 1B). While previous studies have established the structural flexibility of pillarplexes (also in a biological context),^[^ ^30^, ^31^, ^32^ ^]^ we now report additional metal‐ligand coordination as a driving force to overcome a size/shape mismatch between host and guest, enabling the binding of a hydrophilic model substrate (tetrahydrofuran, THF). Furthermore, the choice of Cu^I^ ions conceptually enables redox activity of the cavitand, thereby fulfilling all three key properties of artificial metalloenzyme‐like receptors for their future possible use in catalysis and sensing. Detailed insights into the substrate insertion process and a preliminary evaluation of the receptor's metal‐based redox activity are provided.

## 2 Results and Discussion

**Figure 2:**
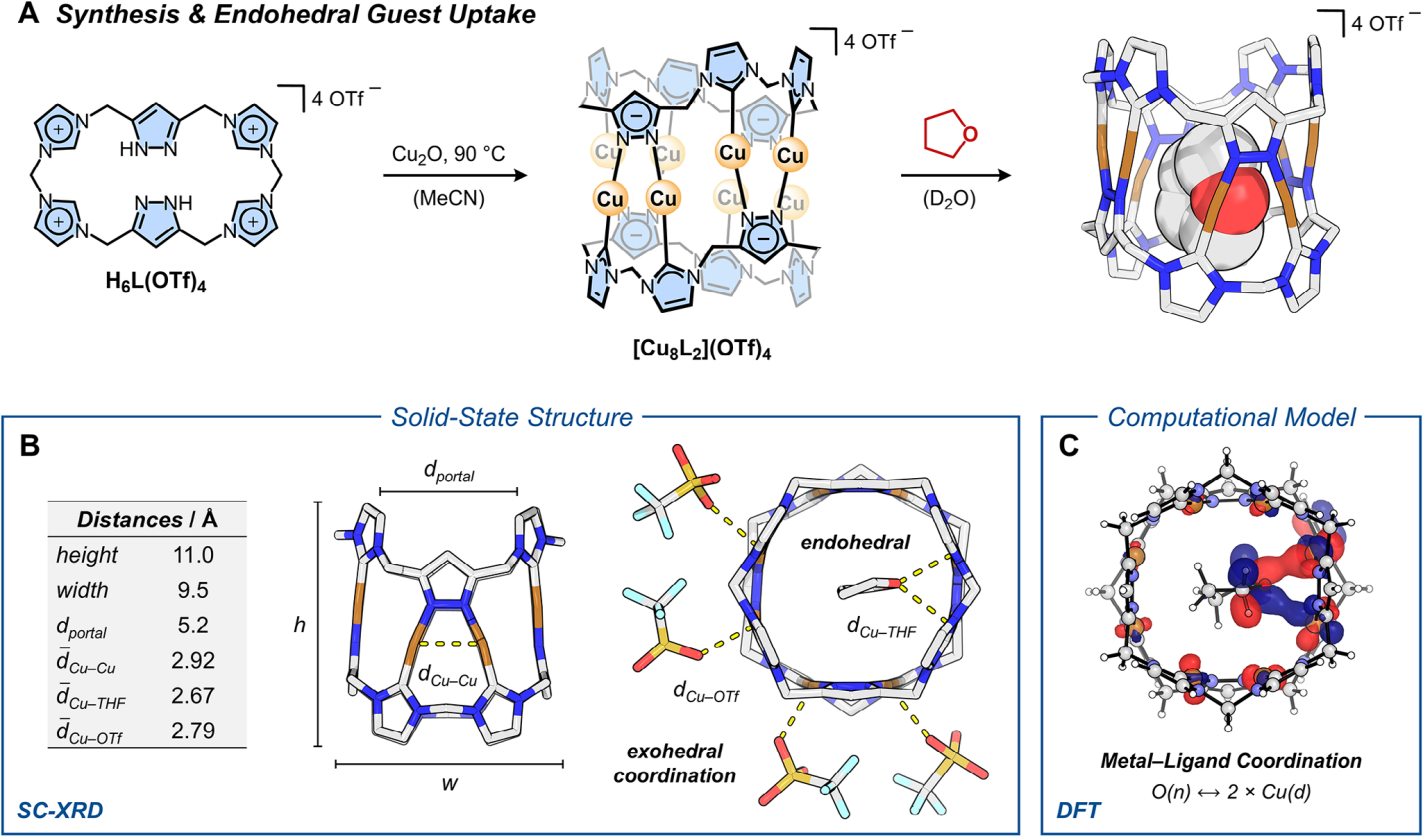
(**A)** Synthesis of **[Cu_8_L_2_](OTf)_4_** and guest insertion of (THF) into the pillarplex cavitand. The SC‐XRD structure of the host‐guest assembly is illustrated on the right, with the **[Cu_8_L_2_]^4+^** cation shown in capped‐sticks representation and THF as *van der Waals* spheres. *Color code*: gray (C), blue (N), red (O), turquoise (F), dark yellow (S), bronze (Cu). Counter ions and solvent molecules are omitted for clarity; (**B)**. SC‐XRD structure of **[Cu_8_L_2_](OTf)_4_**, highlighting endo‐ and exohedral Cu─O coordination by THF and triflate (OTf), respectively. Some counter ions and solvent molecules are omitted for clarity. Structural parameters such as the height (*h*) and width (*w*) of the metallocavitand are summarized in the grey box (*d*_Cu‐Cu_: average intermetallic distance within a Cu_2_ fragment bound to the same pyrazolate ligand, *d*_Cu–THF_: average Cu─O distance to THF, *d*_Cu–OTf_: average Cu─O distance to OTf ions); (**C**) Kohn‐Sham orbital (HOMO–29) of the insertion compound **THF** ⊂ **[Cu_8_L_2_]^4+^**, calculated at the ωB97X‐V/def2‐TZVP level of theory in the gas phase, supporting the presence of endohedral Cu─O coordination.

Pillarplex **[Cu_8_L_2_](OTf)_4_** was prepared by deprotonation of the calix[4]imidazolium[2]pyrazole ligand precursor **H_6_L(OTf)_4_**, using Cu_2_O as an intrinsically basic Cu^I^ source (Figure 2A). This approach afforded significantly cleaner product than the use of other Cu^I^ precursors, such as CuOAc or [Cu(MeCN)_4_]PF_6_, in conjunction with external bases. The ^1^H NMR spectrum of **[Cu_8_L_2_](OTf)_4_** shows a pattern consistent with previously reported pillarplexes (see Supporting Information, Figure S1),^[^ ^29^, ^33^ ^]^ and a ^13^C NMR signal at 177.3 ppm characteristic of Cu^I^‐bound carbene carbons^[^ ^34^ ^]^ corroborates the formation of the target compound. Elemental analysis supported the proposed composition, and high‐resolution mass spectrometry (HR‐HESI‐MS) confirmed the presence of **[Cu_8_L_2_](OTf)*_n_* ^(4–^ *^n^* ^)+^** ions (*n* = 0–3) (see Supporting Information, Figure S22).

Single‐crystal X‐ray diffraction (SC‐XRD) unambiguously proved that **[Cu_8_L_2_](OTf)_4_** adopts a cavitand structure capable of hosting small molecules (Figure 2B), confirming previous structural proposals.^[^ ^35^, ^36^, ^37^ ^]^ In our prior work, we demonstrated that guest uptake is strictly limited to the size and shape of the pillarplex pore.^[^ ^29^, ^30^, ^31^, ^38^ ^]^ While linear alkanes readily form host‐guest assemblies, larger cyclic guests such as benzene are too bulky to insert into the narrow pillarplex cavity. In general, guest uptake can be rationalized by shape complementarity of the pillarplex cavity and the guest molecule. These are assessed by comparing the pillarplex pore opening (*d*_pore_) and inner diameter (*d*_inner_) with the lateral diameter of the guest (see Supporting Information, Figure S28). For example, 1,12‐diaminododecane shows a very high affinity toward host‐guest assembly in polar solvents (e.g., acetonitrile or water),^[^ ^29^, ^39^ ^]^ which correlates reasonably well with a small lateral diameter (*d*_alkane_ ≈ 4.2 Å) that fits the larger pore opening (*d*_pore_ ≈ 5.2 Å) and is still compatible with the slightly smaller inner diameter (*d*_inner_ ≈ 3.7 Å) of pillarplex **[Cu_8_L_2_](OTf)_4_**. In stark contrast, we have now discovered the insertion of a shape‐incompatible, cyclic molecule into **[Cu_8_L_2_](OTf)_4_**. The SC‐XRD structure of the organometallic nanotube clearly reveals its cavity to be occupied by tetrahydrofuran (THF), introduced during single‐crystal growth. Specifically, THF served as an anti‐solvent, slowly diffusing into an acetonitrile solution of the pillarplex. With a much larger lateral diameter (*d*_THF_ = 5.2 Å) than typical linear alkanes, this poses a drastic steric mismatch between the host (*d*_pore_ ≈ 5.2 Å, *d*_inner_ ≈ 3.7 Å) and the cyclic guest. These steric considerations raise the question how the observed host‐guest assembly can be formed and stabilized. In terms of stabilization, a combination of dispersive interactions and metal‐ligand coordination between the host and guest within the cavity can thermodynamically compensate for their steric mismatch (Figure 2B). Specifically, the THF oxygen bridges two adjacent Cu^I^ ions in a *μ* ^2^‐binding mode, with Cu─O_THF_ distances (*d*_Cu–THF_) of 2.591(9) Å and 2.75(1) Å, both significantly shorter than the sum of the *van der Waals* radii for copper and oxygen (*Σr*_vdW_ = 3.48 Å).^[^ ^40^ ^]^ Such *μ* ^2^​‐coordination of THF to a Cu^I^_2_​ pair was previously reported, but with only two crystallographically characterized examples to date remains rare.^[^ ^41^, ^42^ ^]^ The Cu─O_THF_ distances in this study align closely with those observed in THF‐coordinated Cu^I^ alkynyl clusters, strongly suggesting a similar type of metal‐ligand bonding.^[^ ^42^ ^]^ Along this line, for all cuprophilic interactions within **[Cu_8_L_2_](OTf)_4_**,^[^ ^43^ ^]^ the intermetallic distances of Cu–Cu pairs with additional THF coordination are notably shorter (*d*_Cu–Cu,THF_ = 2.8715(9) Å) than those without (*d*_Cu–Cu_ = 2.9664(8) Å). This demonstrates structural flexibility of the Cu–Cu pairs upon uptake of THF by **[Cu_8_L_2_](OTf)_4_**.

For further insight into the guest uptake and host‐guest interactions, gas‐phase DFT calculations of the assembly between THF and **[Cu_8_L_2_]^4+^** were performed.^[^ ^44^ ^]^ In good agreement with the SC‐XRD structure, the computed Cu–Cu distance within the Cu_2_O_THF_ fragment (*d*_Cu–Cu,THF_ = 2.874 Å) is shorter than in the Cu_2_ pairs which lack endohedral coordination (average *d*_Cu–Cu_ = 2.932 Å). Similarly, the calculated Cu─O_THF_ distances (*d*_Cu–O_ = 2.449 Å and 2.631 Å) align reasonably well with the experimental values. A simple visual inspection of the Kohn–Sham orbitals (e.g., HOMO–29, Figure 2C) suggests electronic interactions between the oxygen lone pair of THF and *d*‐orbitals of both Cu^I^ ions. To quantify this, we performed a Quantum Theory of Atoms in Molecules (QTAIM) analysis,^[^ ^45^, ^46^ ^]^ which identified bond critical points (BCPs) between all atoms within the Cu_2_O_THF_ fragment (see Supporting Information, Table S5 and Figure S29). Low electron density (*ρ*) and a slightly positive Laplacian (∇^2^ *ρ*) at these BCPs support Cu─O coordination as well as cuprophilic (Cu─Cu) interactions within the Cu_2_O_THF_ fragment.^[^ ^47^ ^]^ To quantify the stabilization of THF within the cavity of **[Cu_8_L_2_]^4+^**, we examined the thermodynamics of the host‐guest assembly in the gas phase. Consistent with experimental observations, the process was determined to be exergonic (ΔG_R_ = −41 kJ mol^−1^), driven enthalpically (ΔH_R_ = −101 kJ mol^−1^) and counterbalanced by a substantial entropic penalty (*T*ΔS_R_ = −60 kJ mol^−1^). To assess the influence of coordination on the insertion energetics, we also calculated the guest uptake of cyclopentane as a structural analogue of THF without the ability to coordinate to the metal centers. Unlike THF, the incorporation of cyclopentane into **[Cu_8_L_2_]^4+^** was computed to be effectively thermoneutral (ΔG_R_ = −4 kJ mol^−1^) due to a much less favorable enthalpic contribution (ΔH_R_ = −57 kJ mol^−1^) while exhibiting a similar entropic penalty (TΔS_R_ = −53 kJ mol^−1^). These results demonstrate that the endohedral Cu─O coordination between THF and **[Cu_8_L_2_](OTf)_4_** plays a pivotal role in driving guest insertion.

**Figure 3:**
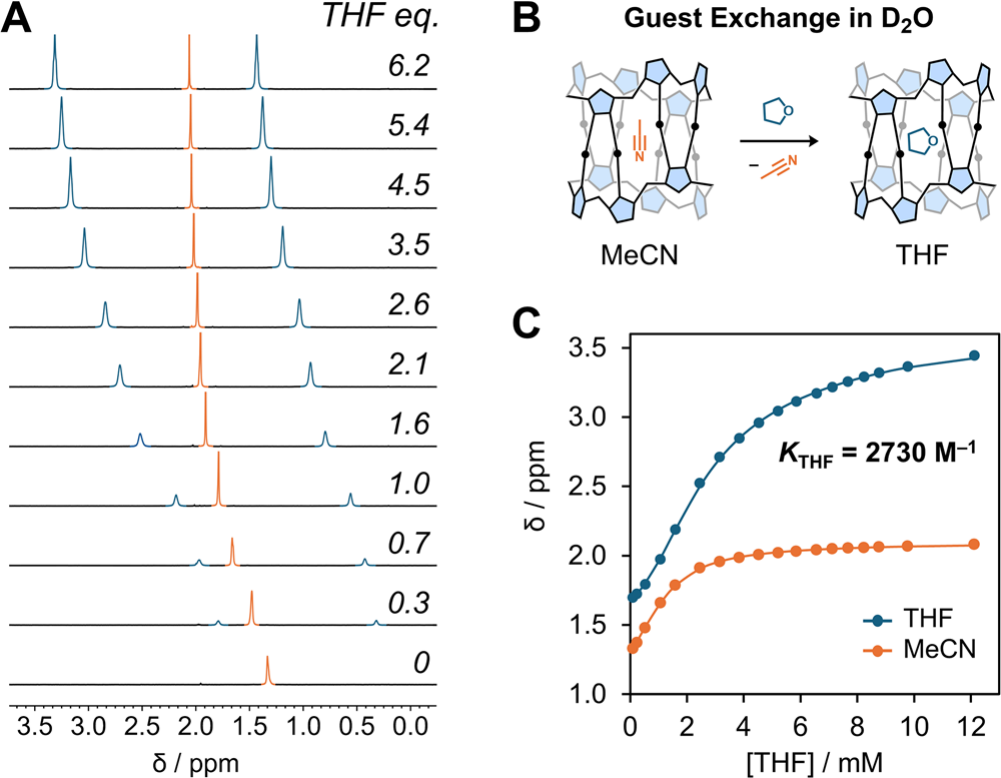
(**A**) ^1^H NMR titration (500 MHz, 294 K) of THF added to **[Cu_8_L_2_](OTf)_4_** (1.57 mM in D_2_O). The NMR resonances of THF and residual MeCN shift downfield with increasing THF concentration, indicating competitive binding with the pillarplex. *Color code*: blue (THF), orange (MeCN); (**B)** Schematic representation of the host‐guest assembly. *Color code*: light blue (pillarplex), blue (THF), orange (MeCN); (**C)** Fitted titration data for the competitive binding of THF and MeCN to **[Cu_8_L_2_](OTf)_4_** (spheres = experimental data, lines = fitted model).

Encouraged by the predicted thermodynamic preference for insertion in the gas phase, we investigated the assembly of THF and **[Cu_8_L_2_](OTf)_4_** in solution *via* ^1^H NMR titration. D_2_O was chosen as a solvent, further facilitating guest insertion by hydrophobic interactions.^[^ ^48^ ^]^ Despite the general sensitivity of Cu^I^ complexes to water and oxygen, **[Cu_8_L_2_](OTf)_4_** demonstrated remarkable stability in aqueous solution under ambient atmosphere, remaining intact for at least 16 hours before significant degradation was observed (see Supporting Information, Figure S7–S9). This set the stage for a systematic study of the binding interactions. Incremental additions of THF to a solution of the Cu^I^ pillarplex (1.57 mM in D_2_O) resulted in pronounced shifts of the guest resonances (see Figure 3A and Supporting Information, Figure S20), reflecting the formation of an insertion complex by penetration of THF deeply into the pillarplex pore. Only a single set of signals was observed for the guest throughout the titration, indicating that the free and bound forms of THF are in fast, dynamic equilibrium on the ^1^H NMR timescale. In contrast, the resonances associated with **[Cu_8_L_2_](OTf)_4_** remained nearly unchanged upon guest addition. The concentration‐dependent chemical shift data throughout the titration were modelled using *Musketeer* (Figure 3C),^[^ ^49^ ^]^ considering the competition between THF and residual MeCN (*cf*. Supporting Information, Section 5) for insertion into the pillarplex cavity (see Supporting Information, Tables S1–2). From this analysis, an association constant *K*_THF_ of 2730 M^−1^ (RMSE: 7.4 × 10^−3^ ppm) was estimated for THF insertion, demonstrating strong binding with **[Cu_8_L_2_](OTf)_4_** in solution. In contrast, the recognition of such a highly hydrophilic, neutral molecule in aqueous solution remains inherently challenging for synthetic receptors.^[^ ^50^ ^]^ For instance, structurally similar tubular‐shaped hosts by Jiang^[^ ^51^ ^]^ and cage‐type hosts by Nitschke^[^ ^52^ ^]^ exhibit considerably lower binding affinities due to the absence of accessible metal sites required for endohedral coordination, in contrast to **[Cu_8_L_2_](OTf)_4_**. In fact, to the best of our knowledge, only Yu and Rebek's “deep cavitand” with its large, shape‐compatible pore has been shown to bind THF more strongly than our platform.^[^ ^50^ ^]^

Collectively, the experimental and theoretical evidence from the solid state, solution, and gas phase strongly supports that the accommodation of THF into the cavity of **[Cu_8_L_2_](OTf)_4_** is exergonic and driven by metal‐ligand coordination.

**Figure 4:**
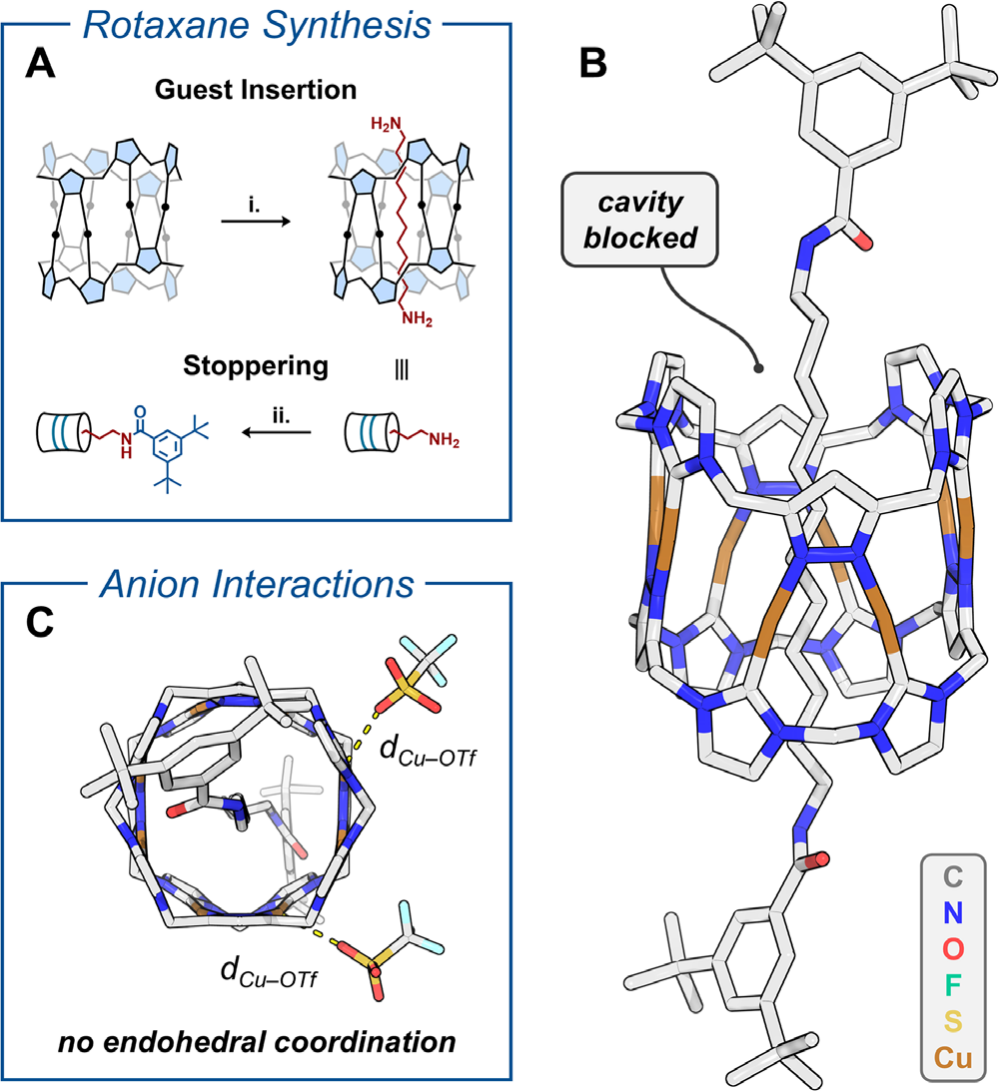
(**A)** Two‐step synthesis of [2]rotaxane **Rot[Cu_8_L_2_](OTf)_4_**: **i**. insertion of 1,12‐diaminododecane into the pore of pillarplex **[Cu_8_L_2_](OTf)_4_**, **ii**. capping of the terminal amines with 3,5‐di‐*tert*‐butylbenzoic anhydride and DIPEA. Pillarplexes are shown schematically (*vide supra*); (**B)** and (**C)** SC‐XRD structure of **Rot[Cu_8_L_2_](OTf)_4_** in capped‐sticks representation, highlighting the exohedral Cu─O coordination of triflate ions. The average Cu─O_OTf_ distance, *d*_Cu–OTf_, is 2.67 Å. Selected counter ions and solvent molecules are omitted for clarity.

Besides endohedral coordination, exohedral metal‐ligand interactions are possible and were observed in the solid state between the triflate ions and the Cu^I^ pillarplex. This is reflected by short Cu─O_OTf_ distances (*d*_Cu–OTf_), ranging from 2.600(5) Å to 2.96(2) Å – only slightly longer than the Cu─O_THF_ bonds (Figure 2). By blocking the cavity of **[Cu_8_L_2_](OTf)_4_** through rotaxane formation, dynamic guest insertion and endohedral coordination is prevented. This reduces the complexity of possible metal‐ligand interactions as the metal centers are only accessible from the outside. Hence, we synthesized rotaxane **Rot[Cu_8_L_2_](OTf)_4_** by following a previously reported protocol (Figure 4A).^[^ ^39^ ^]^ First, Cu^I^ pillarplex **[Cu_8_L_2_](OTf)_4_** was treated with 1,12‐diaminododecane in acetonitrile to form a pseudorotaxane (see Supporting Information, Figures S10–12). The terminal amines were then stoppered *via* amide coupling in presence of 3,5‐di‐*tert*‐butylbenzoic anhydride and DIPEA (*N,N*‐diisopropylethylamine), affording **Rot[Cu_8_L_2_](OTf)_4_** in near‐quantitative yield (Figure 4A). HR‐HESI‐MS strongly supported the presence of the mechanical bond, as shown by the detection of **Rot[Cu_8_L_2_(OTf)*_m_*]^(4–^ *^m^* ^)+^** (*m* = 0–2) ions (see Supporting Information, Figure S23). The structure of the interlocked molecule was confirmed by SC‐XRD (Figure 4B), revealing exohedral Cu─O coordination between Cu^I^ and OTf ions. Similar to **[Cu_8_L_2_](OTf)_4_**, short Cu─O_OTf_ distances (*d*_Cu–OTf_) of 2.607(4) Å and 2.740(3) Å were found.

**Figure 5:**
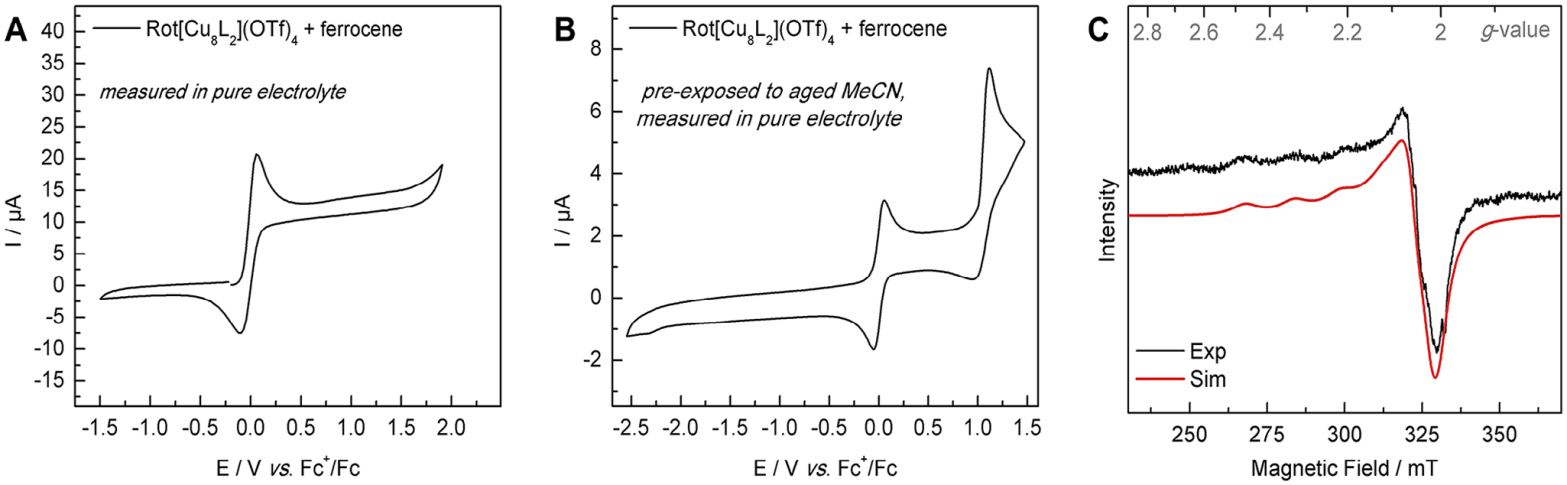
(**A)** CV of **Rot[Cu_8_L_2_](OTf)_4_** measured in a 0.1 M [Me_4_N]PF_6_ electrolyte solution prepared with pure MeCN solvent with ferrocene (ca. 1 mg) added for referencing (scan rate 50 mV/s, 2 mm GC disk working, Pt counter and Ag wire pseudo‐reference electrode). (**B)** CV of **Rot[Cu_8_L_2_](OTf)_4_** previously exposed to aged MeCN and ferrocene (ca. 0.25 mg), measured under otherwise identical conditions as in subfigure A; (**C)** X‐Band EPR spectrum of 12 mM **Rot[Cu_8_L_2_](OTf)_4_** (pre‐exposed to aged MeCN) in 0.1 M [Me_4_N]PF_6_ in MeCN solution after electro‐oxidation (*ν* = 9.267 GHz, *P* = 5.0 mW, modulation width = 0.4 mT, *T* = 133 K). The experimental data (black) was best fitted (red) with *g*_1_ = 2.279, *g*_2_ = 2.058, *g*_3_ = 2.027 and line widths of W_1_ = 5.5 mT, W_2_ = 3.9 mT, and W_3_  =  3.9 mT, a coupling constant A_1_ = 44 mT on *g*_1_, and unresolved hyperfine coupling on *g*_2_ and *g*_3_.

Up to this point, we have demonstrated the geometric flexibility and coordination ability of Cu‐based pillarplexes. An additional key property of metalloenzyme receptors that inspired this study is redox activity. To introduce this crucial property in pillarplex cavitands for the first time, Cu^I^ ions were deliberately used to construct the supramolecular complexes, rather than previously employed redox‐inert Ag^I^ ions. CV experiments were performed as the final part of the study to evaluate whether the copper centers indeed induce redox activity to the system. CV responses of transition metal complexes can be highly sensitive to solvent coordination and dissociation, as well as to interactions with electrolyte ions. Considering the ability of pillarplex **[Cu_8_L_2_](OTf)_4_** to support such interactions, both inside its pore and on its outer surface, these interactions and resulting guest‐specific pore breathing effects are likely to add complexity to the obtained data and its interpretability. In response, we kept interactions inside the pore constant by choosing rotaxane **Rot[Cu_8_L_2_](OTf)_4_** for CV experiments. In pure anhydrous MeCN with 0.1 M [Me_4_N]PF_6_ supporting electrolyte and ferrocene added for internal referencing, no redox event was observed for **Rot[Cu_8_L_2_](OTf)_4_** in the range from −1.5 to +2.0 V (Figure 5A). Reasons therefore may include supramolecular interactions, limiting accessibility of principally electro‐active sites,^[^ ^53^ ^]^ and in case of **Rot[Cu_8_L_2_](OTf)_4_**, a potentially expected Cu^II^/Cu^I^ oxidation^[^ ^54^ ^]^ may be inaccessible due to the high accumulated +4 charge of the supramolecular assembly.

Interestingly, for samples of **Rot[Cu_8_L_2_](OTf)_4_** in aged anhydrous MeCN electrolyte, a quasi‐reversible oxidation event is indeed measured at a half‐wave potential of +1.05 V, potentially caused by trigger molecules originating from MeCN degradation (e.g., acetic acid, acetate, or acetamide)^[^ ^55^ ^]^ (see Supporting Information, Figure S24). This hypothesis is supported by two control experiments in which aged acetonitrile was either added to an electrochemically silent sample of **Rot[Cu_8_L_2_](OTf)_4_** in pure MeCN electrolyte (see Supporting Information, Figure S25), or used for pre‐treating a sample measured in pure MeCN (Figure 5B). In both cases, the same quasi‐reversible oxidation at +1.05 V could be observed, confirming that trigger molecules remain at the rotaxane after exposure.

Spectro‐electrochemical EPR analysis of a pre‐treated rotaxane sample unambiguously revealed the formation of Cu^II^ ions upon the observed electro‐oxidation (see Figure 5C and Supporting Information, Figure S27). First, an EPR spectrum of **Rot[Cu_8_L_2_](OTf)_4_** in 0.1 M [Me_4_N]PF_6_ MeCN solution was recorded in an electrochemical EPR cell before applying any current. As expected, the Cu^I^ rotaxane was EPR‐silent. Electrolysis of the sample at room temperature for 30 seconds, followed by flash freezing in liquid nitrogen to preserve all electro‐generated species, afforded an EPR signal of electrochemically oxidized **Rot[Cu_8_L_2_](OTf)_4_**. In agreement with literature examples of Cu^II^ complexes,^[^ ^56^, ^57^, ^58^ ^]^ the EPR signal of the electro‐oxidized species was best simulated as a rhombic spectrum with *g*‐values at *g*_1_ = 2.279, *g*_2_ = 2.058, *g*_3_ = 2.027, and a characteristic hyperfine coupling to the 3/2 nuclear spin of copper with a coupling constant of A_1_  =  44 mT. A ligand‐based redox event is excluded, both by the absence of an organic radical EPR signal and through CV analysis of the analogous redox‐silent Ag^I^ rotaxane, **Rot[Ag_8_L_2_](PF_6_)_4_**, in which Cu^I^ ions are replaced by inert Ag^I^ (see Supporting Information, Figure S26).

## 3 Conclusion

In summary, we present the synthesis and characterization of an organometallic nanotube, **[Cu_8_L_2_](OTf)_4_**, with eight Cu^I^ centers incorporated into its cavitand wall, reminiscent of multinuclear copper enzymes. The structural adaptability of this Cu^I^ receptor enables endohedral coordination of tetrahydrofuran (THF) as a hydrophilic model substrate despite a steric mismatch between the host and guest. SC‐XRD studies and DFT modelling underscore the role of metal‐ligand coordination in driving substrate insertion by *μ*₂‐binding to adjacent Cu^I^ ions within the pillarplex pore. Binding analysis of THF and **[Cu_8_L_2_](OTf)_4_** *via* ^1^H NMR titration shows that the substrate‐receptor complex readily forms in aqueous solution (D_2_O) despite an intrinsically lower solvophobic effect due to the hydrophilicity of the guest. Electrochemical studies on the derived rotaxane **Rot[Cu_8_L_2_](OTf)_4_** revealed that redox activity of the copper ions is sensitive to electrolyte and additives, providing initial insights into the accessibility of the Cu^II^/Cu^I^ redox couple in the pillarplex. Together, the Cu^I^ pillarplex reported herein fulfills key properties of metalloenzyme‐inspired systems: a structurally flexible cavity, substrate uptake governed by endohedral metal‐ligand coordination as well as dispersive interactions, and metal‐based redox activity. With a pore accessible from both cavitand openings, we introduce a novel receptor topology, integrating the reactive metal site directly into the cavitand wall. This paves the way for future in‐depth studies on the reactivity of pillarplex‐based metalloenzyme‐like receptors.

## Supporting Information

The authors have cited additional references within the Supporting Information.^[^ ^59^, ^60^, ^61^, ^62^, ^63^, ^64^, ^65^, ^66^, ^67^, ^68^, ^69^, ^70^, ^71^, ^72^, ^73^, ^74^, ^75^, ^76^, ^77^, ^78^, ^79^, ^80^, ^81^, ^82^, ^83^, ^84^, ^85^, ^86^, ^87^ ^]^

## Conflict of Interests

The authors declare no conflict of interest.

## Supporting information



Supporting Information

## Data Availability

The data that support the findings of this study are available in the supplementary material of this article.
