# Supplementary material for: Endohedral Coordination of Bulky Substrates in Metalloenzyme‐Like Organometallic Nanotubes
Source: Chemistry. 2025 May 3;31(30):e202500775. doi: 10.1002/chem.202500775 (PMC12117177; doi:10.1002/chem.202500775)
Supplement: Supplementary file 1 — Supporting Information [file CHEM-31-e202500775-s001.pdf]

Supporting Information for

Endohedral Coordination of Bulky Substrates in Metalloenzyme-like Organometallic Nanotubes

Thomas Pickl,[a] Patrick Mollik,[a] Markus R. Anneser,[a] Florian Sixt,[a] Korbinian Geißer, [a] Oksana Storcheva,[a] Dominik P. Halter,[a,b] and Alexander Pöthig*[a]*

[a] Catalysis Research Center & TUM School of Natural Sciences, Department of Chemistry, Technical University of Munich, Ernst-Otto-Fischer Str. 1, 85747 Garching, Germany.

[b] Research Group Applied Electrochemistry & Catalysis (ELCAT), Faculty of Applied Engineering, Department of Biochemical and Chemical Engineering, University of Antwerp, Universiteitsplein 1, 2610 Antwerp, Belgium.

Correspondence to: alexander.poethig@tum.de; dominik.halter@tum.de

TABLE OF CONTENTS

1. General Information 1
2. Analytical Methods 1
3. Experimental Procedures 3
   $[Cu_8L_2](OTf)_4$ 3
   Rot$[Cu_8L_2](OTf)_4$ 4
4. NMR Spectra 5
   $[Cu_8L_2](OTf)_4$ 5
   Stability Assessment of THF-inserted $[Cu_8L_2](OTf)_4$ in D_2O 7
   1,12-Diaminododecane$\subset[Cu_8L_2](OTf)_4$ 8
   Rot$[Cu_8L_2](OTf)_4$ 10
5. Determination of Affinity Constants 13
6. HR-MS Spectra 17
   $[Cu_8L_2](OTf)_4$ 17
   Rot$[Cu_8L_2](OTf)_4$ 18
7. Cyclic Voltammetry 19
8. EPR Spectroscopic Details 21
9. Crystallographic Details 22
   $[Cu_8L_2](OTf)_4$ 22
   Rot$[Cu_8L_2](OTf)_4$ 23
   1,12-Diaminododecane 24
   Determination of Structural Parameters 26
10. Computational Details 27
11. References 37

1. General Information

All syntheses were performed under an argon atmosphere (4.6, *Westfalen*) using standard Schlenk techniques or within an *MBraun* Labmaster Pro SP glovebox. HPLC-grade acetonitrile (MeCN), diethyl ether (Et_2O) and tetrahydrofuran (THF) were purchased from *Sigma Aldrich*, dried using an *MBraun* solvent purification system, degassed, and stored over activated molecular sieves (3Å). MeCN-d_3 (dried similarly to the HPLC solvents) and D_2O were obtained from *Eurisotop* and *Sigma Aldrich*, respectively, and used without further purification. **$H_6L(OTf)_4$**, **Rot[Cu_8L_2]$(OTf)_4$** and 3,5-di-*tert*-butylbenzoic anhydride were prepared according to previously reported procedures.[1-2] 1,12-diaminododecane was purchased from Sigma Aldrich and purified by sublimation (85°C, 0.05 mbar) prior to use. *N*,*N*-Diisopropylethylamine was purchased from *TCI* and degassed by argon sparging prior to use. $[Me_4N]PF_6$ was purchased from *TCI* (purity: 99.9% according to the Certificate of Analysis) and used without further purification. All other commercially available chemicals were purchased from commercial vendors and used without further purification.

2. Analytical Methods

NMR spectra were recorded on a *Bruker* AV-500, AVHD-400, AVHD-500 spectrometer or an AV-III-500 spectrometer equipped with a QNP cryo probe, with MeCN-d_3 or D_2O as solvents. Chemical shifts δ are given in parts per million (ppm) and scalar coupling constants ^nJ are given in Hertz (Hz). ^{1}H and ^{13}C NMR chemical shifts recorded in MeCN-d_3 are reported relative to its residual proton signal (^{1}H: δ = 1.94 ppm, ^{13}C: δ = 1.32 ppm) with respect to tetramethylsilane. ^{19}F NMR shifts are reported relative to trichlorofluoromethane. For measurements in D_2O, a capillary containing benzene-d_6 (^{1}H: δ = 7.16 ppm) was added as an external standard and the ^{1}H NMR spectra were referenced to the residual proton signal of benzene-d_6 with respect to tetramethylsilane. Diastereotopic protons are marked with an apostrophe (*e.g.* H_b and $H_{b'}$). The multiplicity of ^{13}C nuclei was determined by a phase-sensitive HSQC experiment where methyl and methine signals are shown in blue and methylene signals are coloured red. HMBC spectroscopy allowed for the assignment of the remaining quaternary carbons.

HR-MS (HESI$^+$) spectra were recorded on a *Thermo Fisher* Exactive Plus Orbitrap mass spectrometer equipped with a *Thermo Fisher* ESI source. Isotopic fine structures were calculated using the web-interface of the R package *enviPat*.[3]

Elemental analyses were performed by the Elemental Analysis Laboratory of the Catalysis Research Center at the Technical University of Munich on a *Eurovector* EA3000 CHNS combustion analyzer. Approx. 1 mg of sample was weighed on a Sartorius CP2P microbalance (± 1 µg resolution), then dynamically spontaneously combusted in a tin boat at 1800 °C, separated by gas chromatography, and detected with a thermal conductivity detector. For calibration, two NIST-certified reference materials were used: sulfanilamide and 2,5-bis-(5-*tert*-butyl-2-benzoxazol-2-yl)-thiophenone (BBOT).

Electrochemical experiments were performed using a *Metrohm* Autolab PGSTAT302N potentiostat for cyclic voltammetry, and a *PalmSens4* potentiostat for EPR spectro-electrochemistry. Glassy carbon (GC) working electrodes were freshly polished with a 0.3µm alumina /

water slurry followed by 1 min sonication in H_2O and then acetone and final blow drying or air drying. Cyclic voltammetry was carried out under argon atmosphere, using a 2 mm glassy carbon disc electrode (working electrode), a 3 mm platinum disc electrode (counter electrode) and a silver rod (pseudo reference electrode) with recrystallised ferrocene (twice from hexane) added for referencing. The electrolyte was a 0.1 M $[Me_4N]PF_6$ (tetramethylammonium hexafluorophosphate) solution in MeCN. Influence of MeCN solvent quality on the electrochemical response was observed. Specifically, pure MeCN was obtained from freshly opened HPLC-grade bottles or from batches purified by factional distillation over CaH_2,[4] as confirmed by electrochemical testing with benzoquinone and phenylenediamine as indicators.[5] Karl-Fischer titration confirmed a residual water content < 3 ppm in fractionally distilled MeCN and < 25 ppm in MeCN from freshly opened HPLC-grade bottles. In this pure electrolyte, samples of **Rot[Cu_8L_2](OTf)$_4$** were electrochemically silent. In samples of aged MeCN that was still anhydrous but had been stored under argon for several months, samples of **Rot[Cu_8L_2](OTf)$_4$** were electrochemically active, likely due to interaction with MeCN decomposition products, including acetamide, ammonia, ammonium acetate, or acetic acid. Samples of **Rot[Cu_8L_2](OTf)$_4$** that are immediately electrochemically active also in pure MeCN electrolyte were obtained by dissolving **Rot[Cu_8L_2](OTf)$_4$** in a minimal amount of aged MeCN, followed by removal of the solvent *in vacuo*, upon which dry powdered sample of pre-exposed **Rot[Cu_8L_2](OTf)$_4$** are obtained for further experiments. Electrolysis for EPR spectro-electrochemistry measurements was performed with gold wire electrodes (working and counter electrodes) and a silver wire pseudo reference electrode inside an EPR spectro-electrochemical cell under argon. Initially, a cyclic voltammogram was measured to ensure setup and sample integrity, as well as to determine the peak potential for **Rot[Cu_8L_2](OTf)$_4$** oxidation against the pseudo reference. At an electrolysis potential that was 100 mV more positive than the oxidation peak potential, the sample (12 mM of **Rot[Cu_8L_2](OTf)$_4$** that was pre-exposed to aged MeCN in a 0.1 M solution of $[Me_4N]PF_6$ in pure MeCN) was then electrolysed inside the EPR tube at room temperature for 30 seconds, towards the end of which the tube was flash frozen in liquid nitrogen, to preserve all electro-generated species for subsequent analysis by EPR spectroscopy.

EPR spectroscopy was performed on a *Jeol* JES-FA 200 spectrometer equipped with a liquid nitrogen cryostat at 133 K and the spectra were simulated using the program W95EPR.[6]

3. Experimental Procedures

$[Cu_8L_2](OTf)_4$

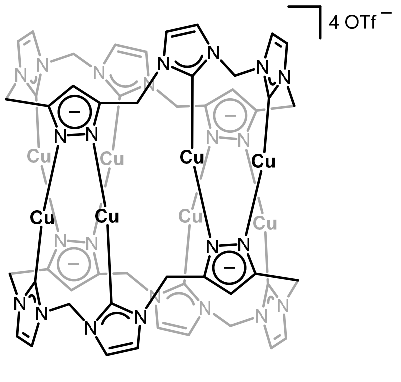

$[Cu_8L_2](OTf)_4$

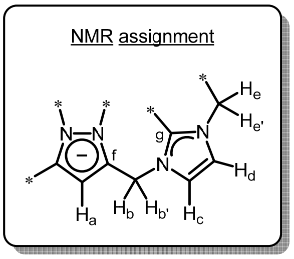

Under an inert atmosphere, ligand precursor **$H_6L(OTf)_4$** (100 mg, 92.5 µmol, 1.0 eq.) and copper(I) oxide (100 mg, 699 µmol, 7.6 eq.) were weighed into a 10 mL Schlenk tube and suspended in 10 mL freshly degassed, dry acetonitrile. The reaction mixture was stirred at 90 °C (oil bath temperature) for 16 h, then cooled to ambient temperature and filtered to remove excess Cu_2O. Purification of **$[Cu_8L_2](OTf)_4$** posed a challenge due to the concomitant formation of NMR-silent, coloured copper(II) impurities. These impurities were removed by concentrating the yellow filtrate to dryness *in vacuo* and washing several times with cold MeCN. After drying *in vacuo*, **$[Cu_8L_2](OTf)_4$** was isolated as a white solid in 45% yield (43 mg, 20.9 µmol). Additional product precipitated from the acetonitrile filtrates at –40 °C under an inert atmosphere and was recovered by filtration.

^{1}H NMR (500.1 MHz, MeCN-d_3, 298 K): δ [ppm] = 7.53 (s, 8H, H_d), 7.46 (s, 8H, H_c), 6.72 (d, ^2J = 15.1 Hz, 4H, H_e), 6.60 (s, 4H, H_a), 5.98 (d, ^2J = 15.1 Hz, 4H, $H_{e'}$), 5.72 (d, ^2J = 15.5 Hz, 8H, H_b), 5.24 (d, ^2J = 15.5 Hz, 8H, $H_{b'}$).

^{13}C NMR (125.8 MHz, MeCN-d_3, 300 K): δ [ppm] = 177.3 (C_g), 150.4 (C_f), 123.8 (C_c), 123.2 (C_d), 122.0 (q, $^1J_{C-F}$ = 319 Hz, CF_3), 104.4 (C_a), 64.1 (C_e), 49.1 (C_b).

^{19}F NMR (376.5 MHz, MeCN-d_3, 300 K): δ [ppm] = –79.3 (SO_3CF_3).

HR-MS (ESI$^+$, MeCN) *m/z* 365.9618 (calc. 365.9623 for $[Cu_8L_2]^{4+}$), 538.2660 (calc. 538.2666 for $[Cu_8L_2(OTf)]^{3+}$), 880.8759 (calc. 880.8771 for $[Cu_8L_2(OTf)_2]^{2+}$), 1912.7062 (calc. 1912.7050 for $[Cu_8L_2(OTf)_3]^{+}$).

EA (%) for **$[Cu_8L_2](OTf)_4$** · MeCN: calc. C 30.85, H 2.25, N 16.65, S 6.10; found C 31.16, H 2.37, N 16.19, S 6.40. Even after prolonged times of drying *in vacuo*, ~ 1 equiv. of MeCN could not be removed and remained in the sample.

Rot[Cu_8L_2](OTf)$_4$

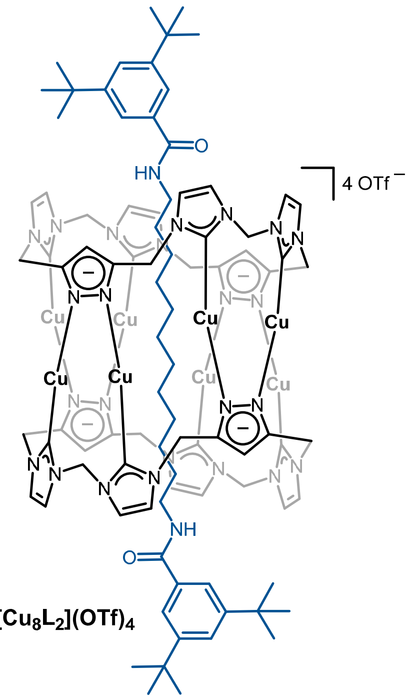

Rot[Cu_8L_2](OTf)$_4$

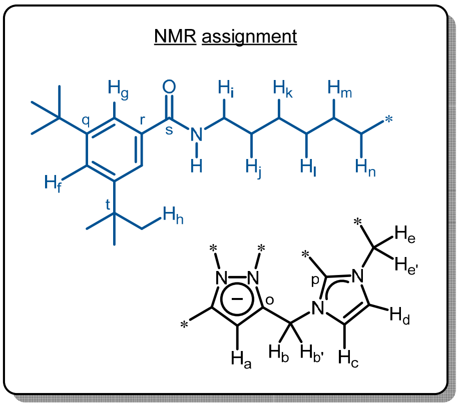

Under an inert atmosphere, pillarplex **[Cu_8L_2](OTf)$_4$** (20.0 mg, 9.70 µmol, 1.0 eq.) and 1,12-diaminododecane (5.0 mg, 24.9 µmol, 2.6 eq.) were weighed into a 10 mL Schlenk tube and suspended in 3 mL freshly degassed, dry acetonitrile. After stirring for 30 min, *N*,*N*-diisopropylethylamine (8.8 µL, 50.4 µmol, 5.2 eq.) and subsequently 3,5-di-*tert*-butylbenzoic anhydride (22.7 mg, 50.4 µmol, 5.2 eq.) were added. The mixture was stirred for 16 h, then cold Et_2O (10 mL) was added to precipitate a white solid. The crude product was washed with more Et_2O and dried *in vacuo* to afford **Rot[Cu_8L_2](OTf)$_4$** as a white solid in 96% yield (25.0 mg, 9.28 µmol).

^{1}H NMR (500.1 MHz, MeCN-*d*$_3$, 298 K): δ [ppm] = 7.76 (d, ^4J = 1.8 Hz, 4H, H_g), 7.71 (t, ^4J = 1.8 Hz, 2H, H_f), 7.67 (d, ^3J = 1.9 Hz, 8H, H_d), 7.44 (d, ^3J = 1.9 Hz, 8H, H_c), 7.10 (t, ^3J = 5.9 Hz, 2H, NH), 6.75 (d, ^2J = 15.0 Hz, 4H, H_e), 6.62 (s, 4H, H_a), 6.00 (d, ^2J = 15.0 Hz, 4H, $H_{e'}$), 5.73 (d, ^2J = 15.4 Hz, 8H, H_b), 5.22 (d, ^2J = 15.4 Hz, 8H, $H_{b'}$), 2.70 (m, 4H, H_i), 1.42 (s, 36H, H_h), 0.19 (m, 4H, H_j), –0.15 (m, 4H, H_n), –0.93 (m, 4H, H_m), –1.07 (m, 4H, H_k), –1.48 (m, 4H, H_l).

^{13}C NMR (125.8 MHz, MeCN-*d*$_3$, 300 K): δ [ppm] = 177.1 (C_p), 168.3 (C_s), 152.4 (C_q), 149.9 (C_o), 135.9 (C_r), 126.5 (C_f), 124.2 (C_c), 123.6 (C_d), 122.2 (C_g), 122.1 (q, $^1J_{C\text{–}F}$ = 321 Hz, CF_3), 104.7 (C_a), 64.3 (C_e), 49.2 (C_b), 40.7 (C_i), 35.8 (C^t), 33.8 (C_n), 32.3 (C_m), 31.6 (C_h), 31.0 (C_j), 30.4 (H_l), 27.7 (C_k).

^{19}F NMR (470.8 MHz, MeCN-*d*$_3$, 300 K): δ [ppm] = –79.3 (SO_3CF_3).

HR-MS (ESI$^+$, MeCN) *m/z* 524.5933 (calc. 524.5938 for Rot[Cu_8L_2]$^{4+}$), 749.1084 (calc. 749.1093 for Rot[Cu_8L_2(OTf)]$^{3+}$), 1198.1393 (calc. 1198.1402 for Rot[Cu_8L_2(OTf)$_2$]$^{2+}$).

EA (%) for **Rot[Cu_8L_2](OTf)$_4$** · Et_2O: calc. C 42.51, H 4.44, N 13.15; found C 42.58, H 4.64, N 13.04, S 4.01. Even after prolonged times of drying *in vacuo*, complete removal of diethyl ether could not be achieved. As shown by ^{1}H NMR, ~ 1 equiv. of Et_2O remained in the sample.

4. NMR Spectra

[Cu_8L_2](OTf)$_4$

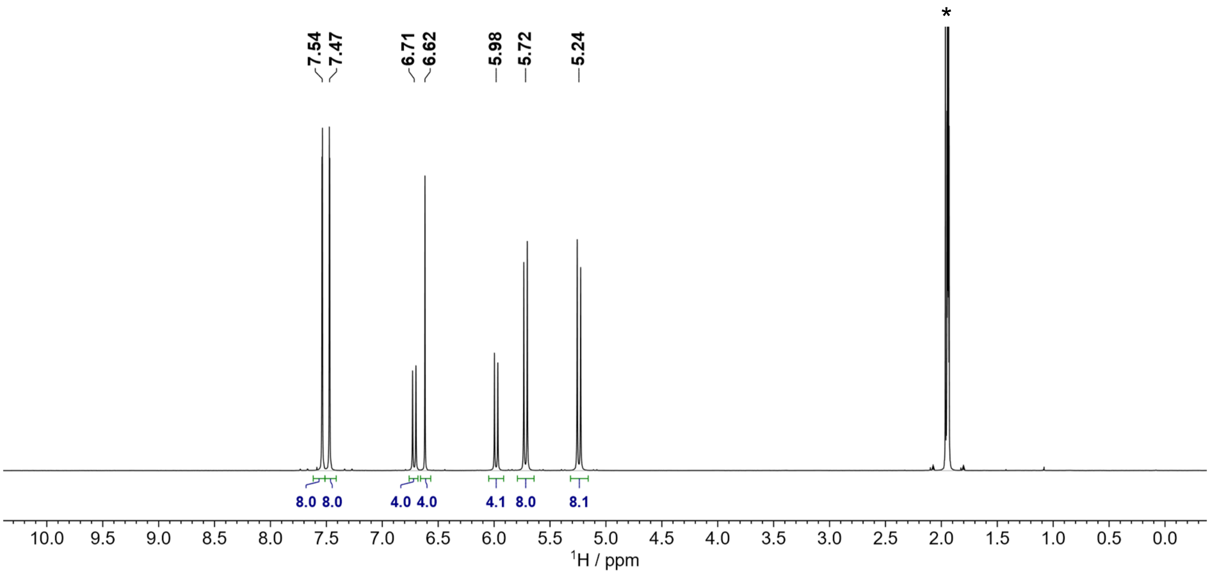

Figure S1 | ^{1}H NMR spectrum (500.1 MHz, 298 K) of **[Cu_8L_2](OTf)$_4$** in MeCN-d_3 (* = residual solvent peak).

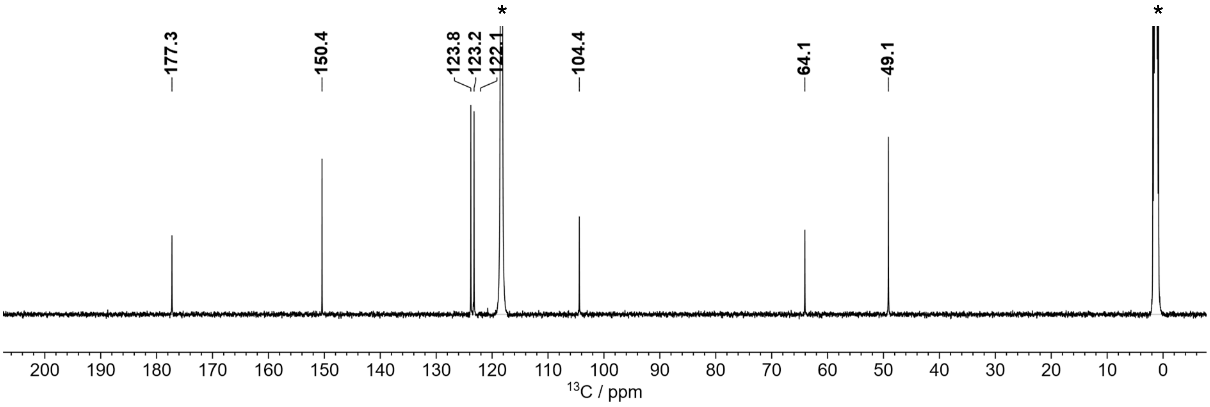

Figure S2 | ^{13}C NMR spectrum (125.8 MHz, 300 K) of **[Cu_8L_2](OTf)$_4$** in MeCN-d_3 (* = residual solvent peaks).

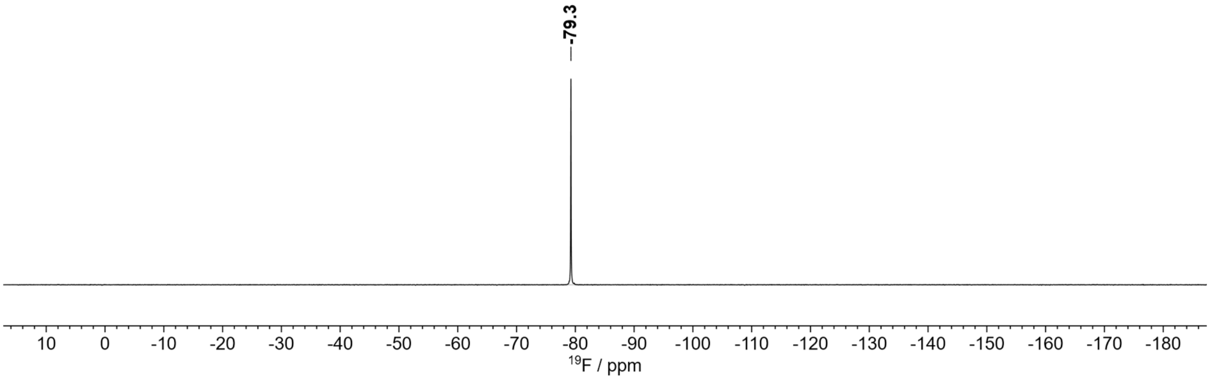

Figure S3 | ^{19}F NMR spectrum (376.5 MHz, 300 K) of **[Cu_8L_2](OTf)$_4$** in MeCN-d_3.

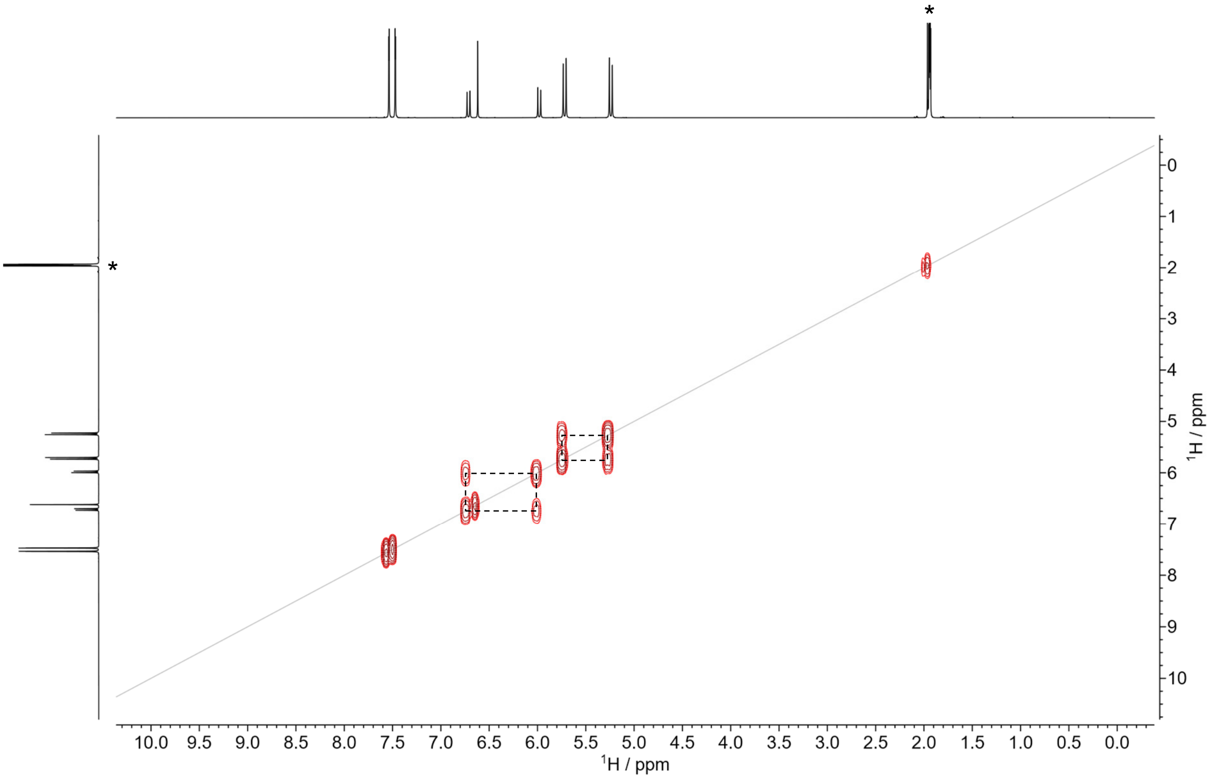

Figure S4 | $^{1}H,^{1}H$ COSY spectrum (500.1 MHz, 298 K) of **[Cu$_8$L$_2$](OTf)$_4$** in MeCN-d_3 (* = residual solvent peaks).

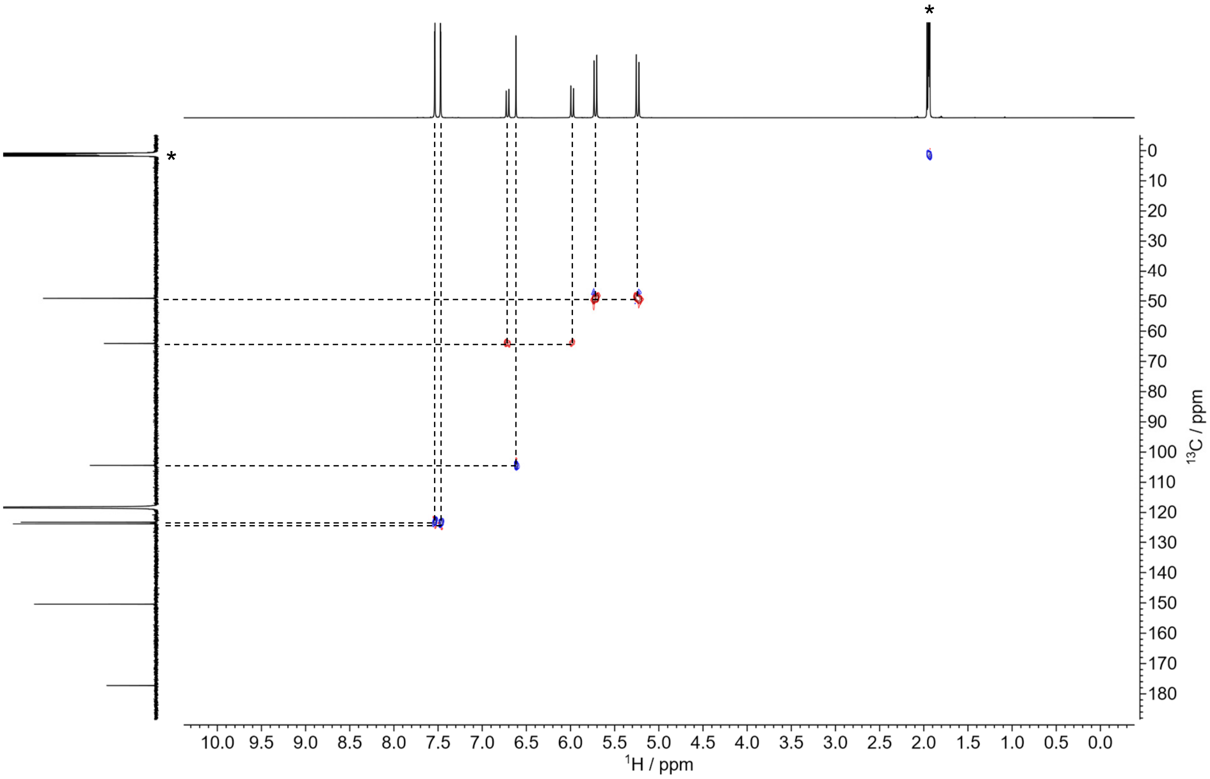

Figure S5 | $^{1}H,^{13}C$ HSQC spectrum (500.1 MHz, 125.8 MHz, 298 K) of **[Cu$_8$L$_2$](OTf)$_4$** in MeCN-d_3 (* = residual solvent peaks).

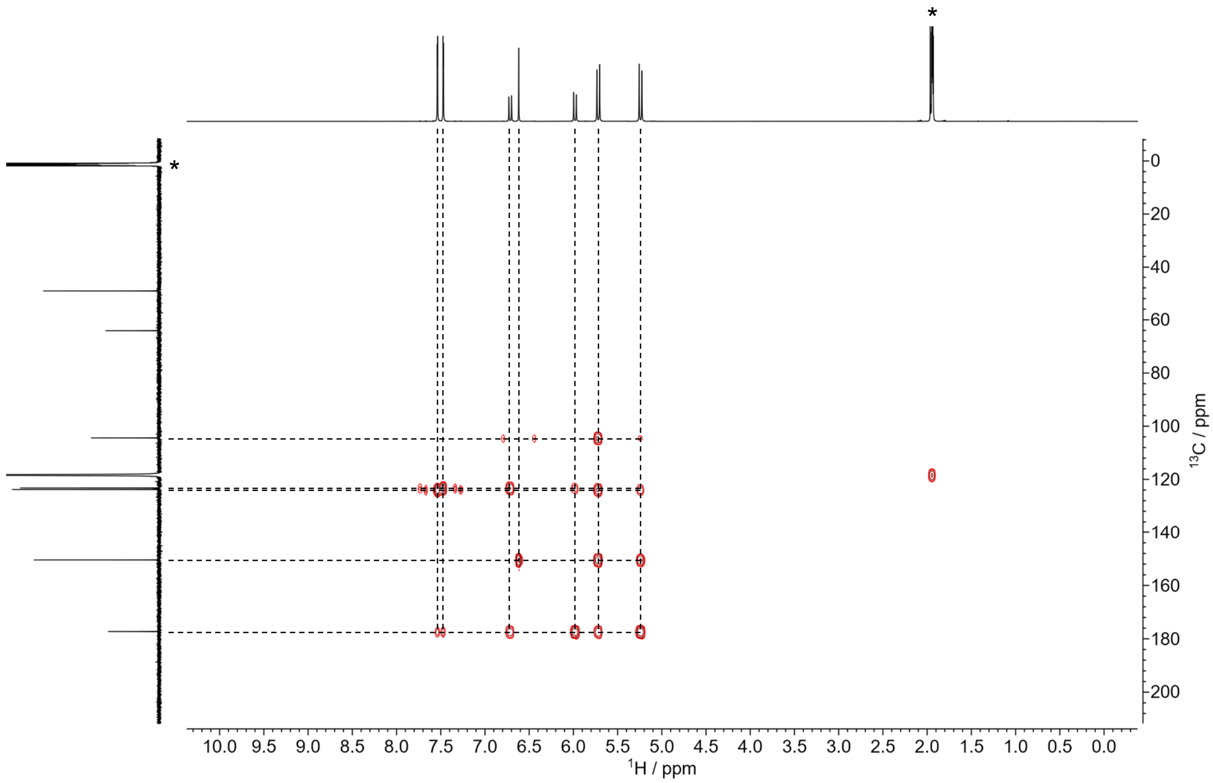

Figure S6 | ^{1}H,^{13}C HMBC spectrum (500.1 MHz, 125.8 MHz, 298 K) of **[Cu_8L_2](OTf)$_4$** in MeCN-d_3 (* = residual solvent peaks).

Stability Assessment of THF-inserted [Cu_8L_2](OTf)$_4$ in D_2O

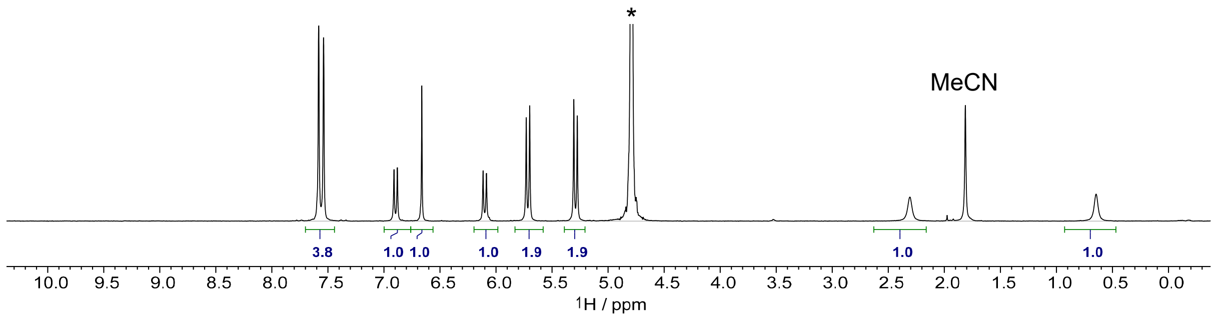

Figure S7 | ^{1}H NMR spectrum (500.1 MHz, 298 K) of **[Cu_8L_2](OTf)$_4$** in D_2O under ambient atmosphere ca. 1 hour after addition (* = residual solvent peak).

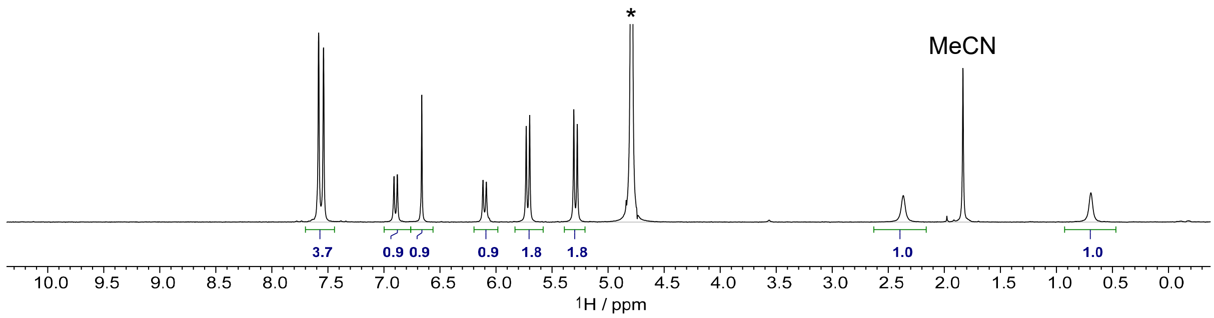

Figure S8 | ^{1}H NMR spectrum (500.1 MHz, 298 K) of **[Cu_8L_2](OTf)$_4$** in D_2O under ambient atmosphere ca. 6 hours after addition (* = residual solvent peak).

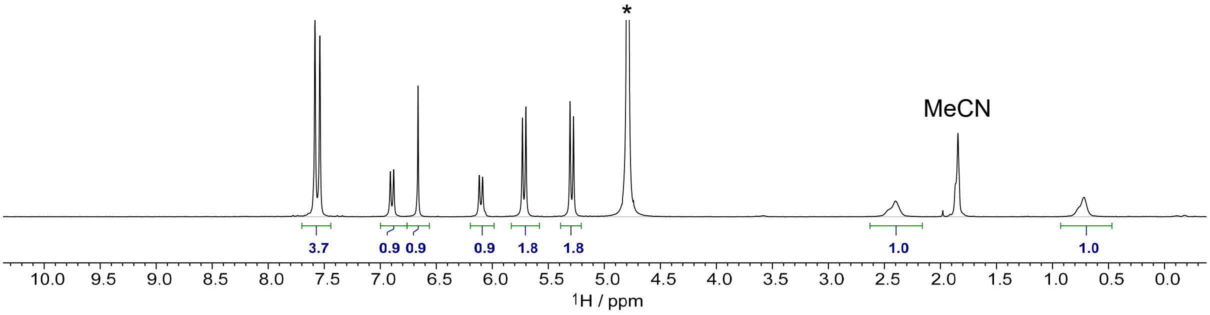

Figure S9 | ^{1}H NMR spectrum (500.1 MHz, 298 K) of **$[Cu_8L_2](OTf)_4$** in D_2O under ambient atmosphere ca. 16 hours after addition (* = residual solvent peak).

Based on the integral ratios of THF (deliberately set to 1) relative to **$[Cu_8L_2](OTf)_4$** (Figures S7–9), it is evident that the pillarplex remains stable in D_2O for at least 6 hours before any signs of degradation become noticeable by ^{1}H NMR. Even after 16 hours, no significant degradation is observed. Nonetheless, all subsequent ^{1}H NMR titration experiments (*cf.* Section 5) were performed within 2 hours of dissolving the compound in D_2O.

1,12-Diaminododecane⊂$[Cu_8L_2](OTf)_4$

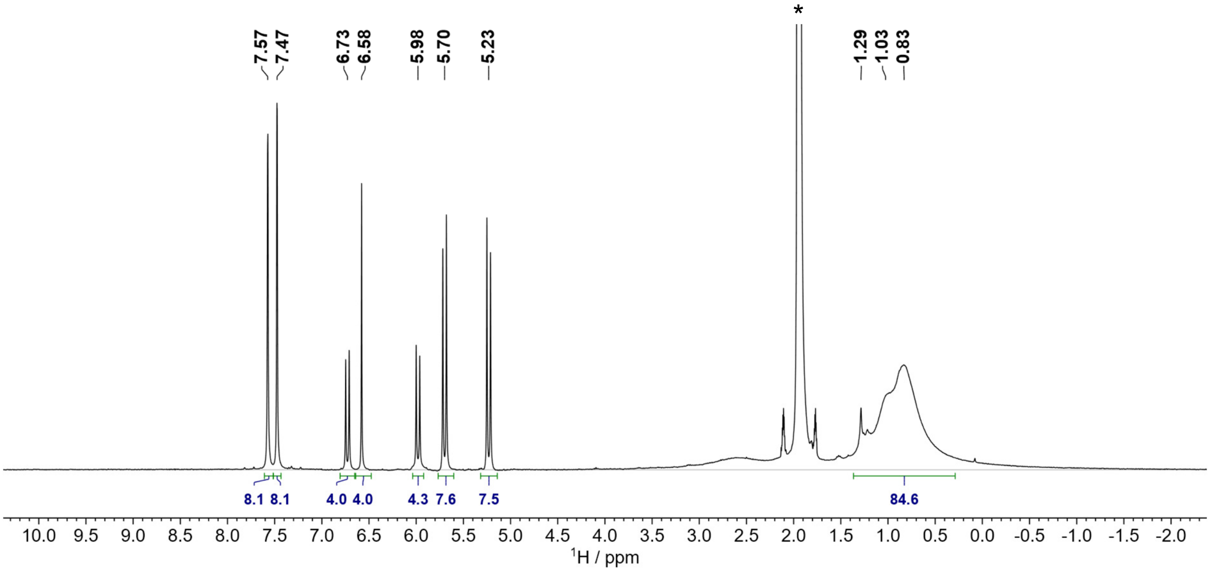

Figure S10 | ^{1}H NMR spectrum (400.1 MHz, 298 K) of the insertion complex obtained after reacting an excess (~3 equiv.) of 1,12-diaminododecane with **$[Cu_8L_2](OTf)_4$** in MeCN-d_3 (* = residual solvent peak). The resonances observed between –0.5 and 1.3 ppm, as well as between 2.5 and 3.0 ppm, were assigned to the diamine inserted in the pillarplex cavity.

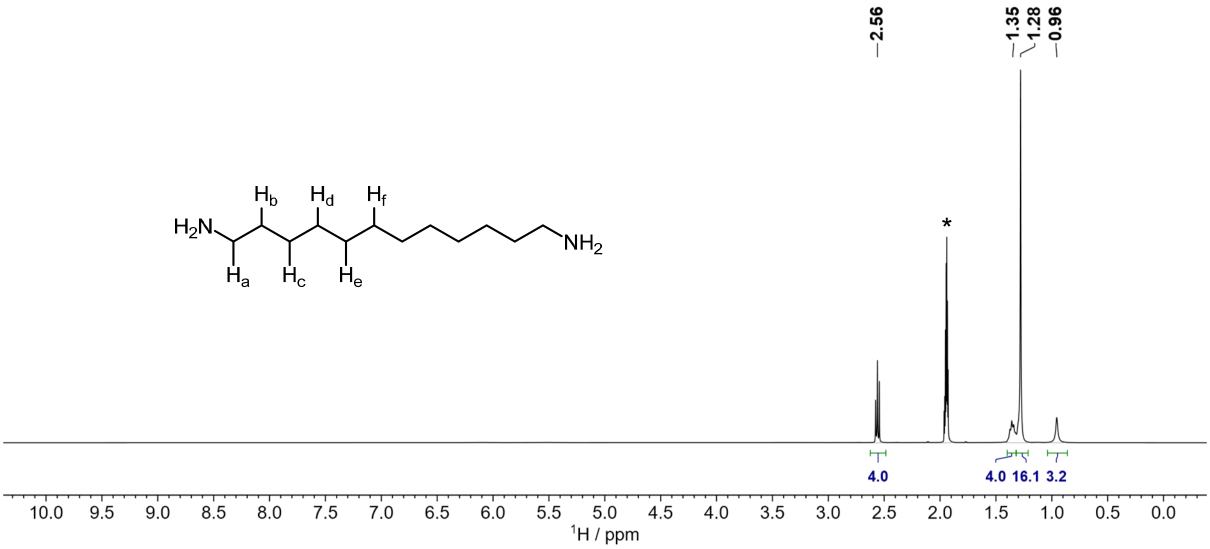

Figure S11 | ^{1}H NMR spectrum (400.1 MHz, 298 K) of a saturated solution of 1,12-diaminododecane in MeCN-d_3 (* = residual solvent peak). Signal assignment: δ [ppm] = 2.56 (t, ^{3}J = 6.7 Hz, 4H, H_a), 1.35 (m, 4H, H_b), 1.28 (s, 16H, $H_{c–f}$), 0.96 (s, 4H, NH_2).

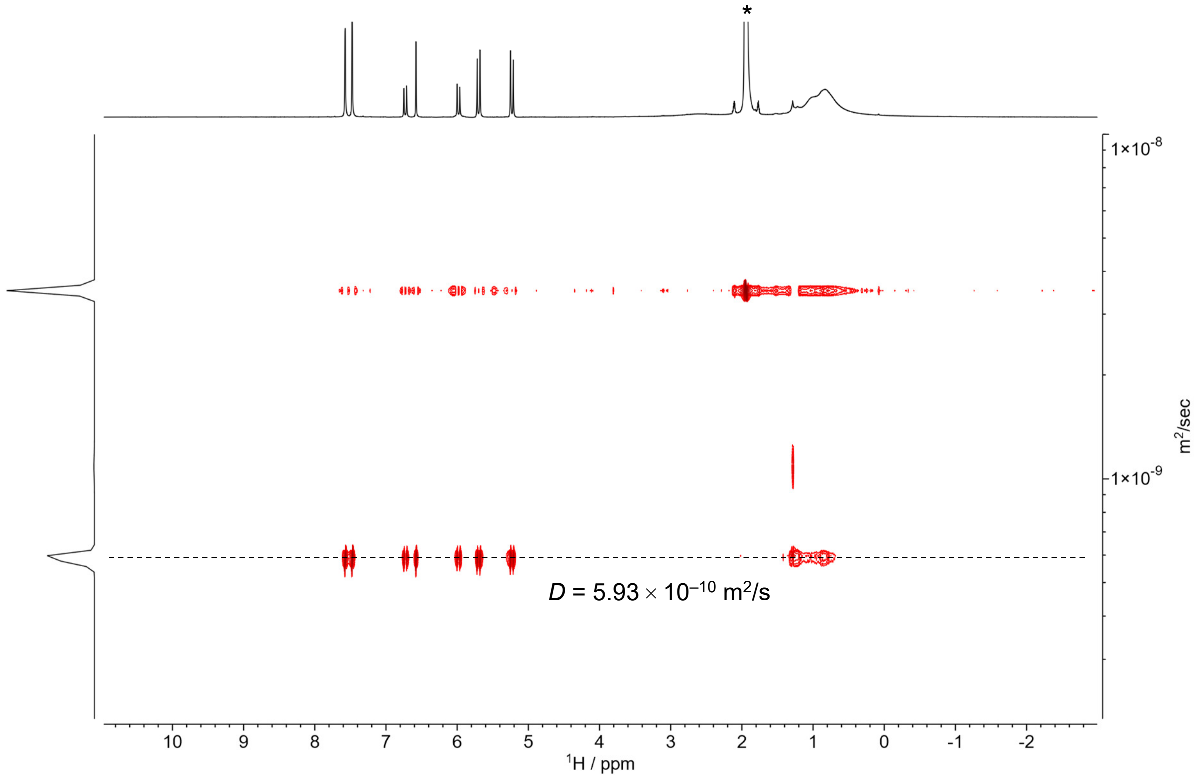

Figure S12 | ^{1}H DOSY spectrum (400.1 MHz, 298 K) of the insertion complex obtained after reacting an excess (~3 eq.) of 1,12-diaminododecane with **$[Cu_8L_2](OTf)_4$** in MeCN-d_3 (* = residual solvent peak). On the timescale of the DOSY experiment, two diffusion coefficients were observed for 1,12-diaminododecane, which were attributed to its dynamic insertion behaviour into the pillarplex pore. Nevertheless, the host and guest also exhibit the same diffusion coefficient D, indicating that they move as a single solute, thereby proving insertion of the alkane.

Rot[Cu_8L_2](OTf)$_4$

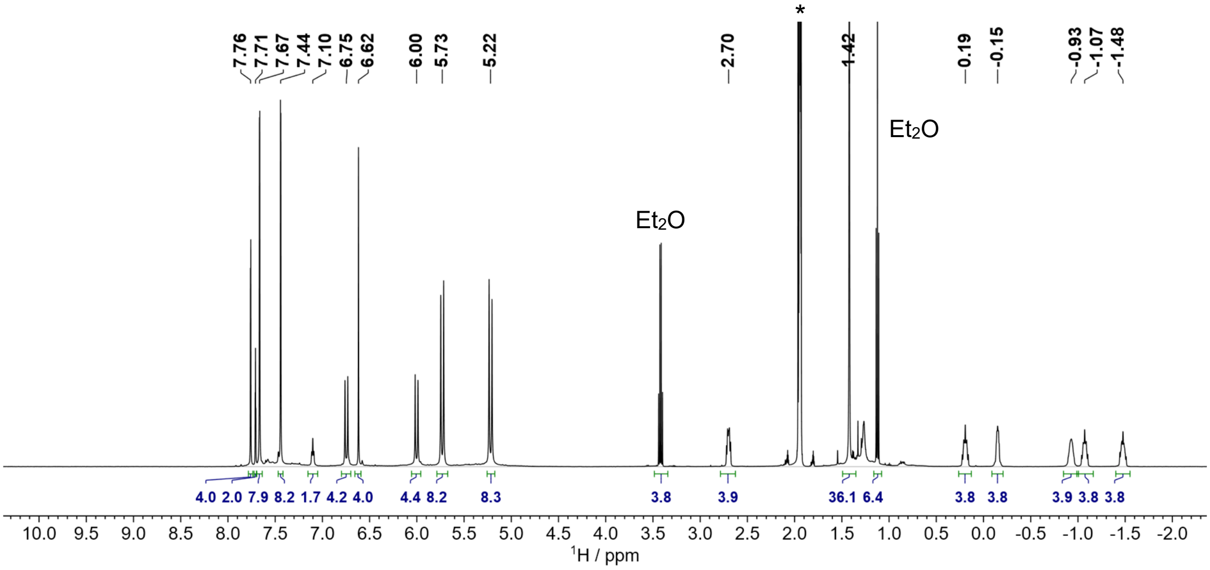

Figure S13 | ^{1}H NMR spectrum (500.1 MHz, 298 K) of **Rot[Cu_8L_2](OTf)$_4$** in MeCN-d_3 (* = residual solvent peak).

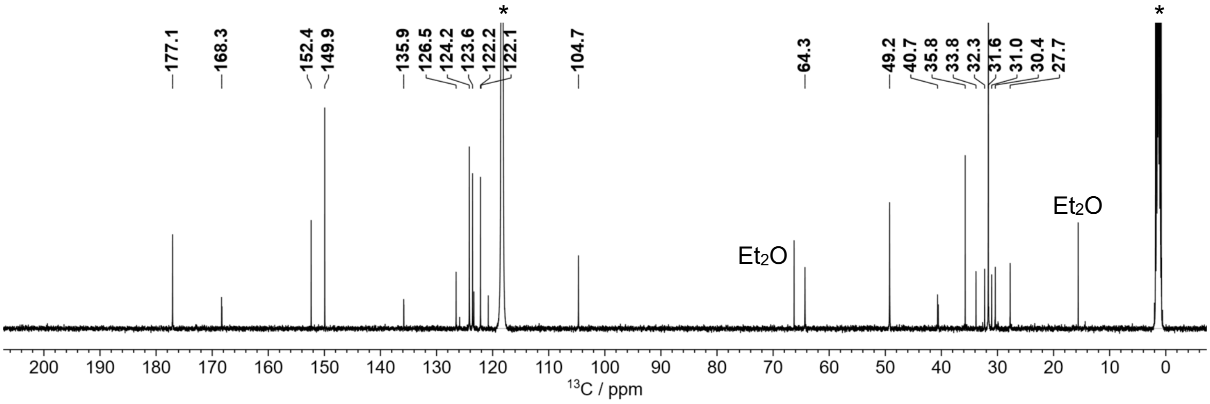

Figure S14 | ^{13}C NMR spectrum (125.8 MHz, 300 K) of **Rot[Cu_8L_2](OTf)$_4$** in MeCN-d_3 (* = residual solvent peaks).

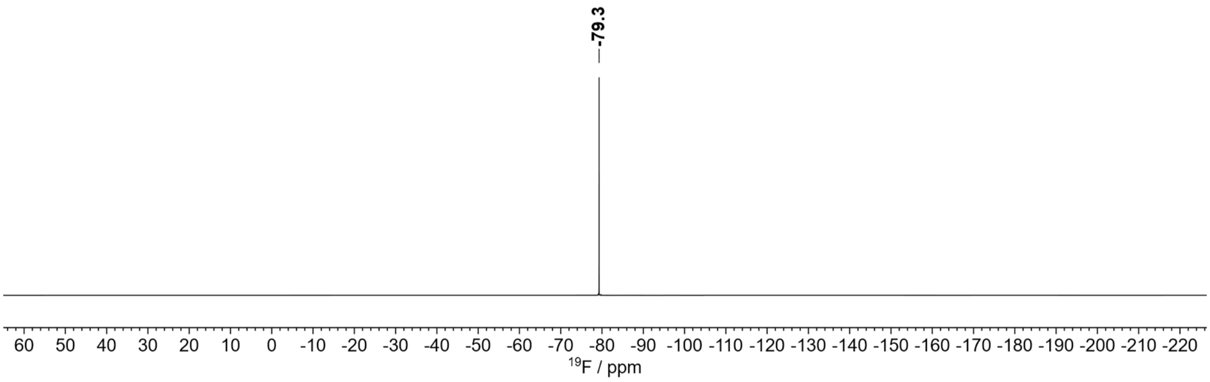

Figure S15 | ^{19}F NMR spectrum (470.8 MHz, 300 K) of **Rot[Cu_8L_2](OTf)$_4$** in MeCN-d_3.

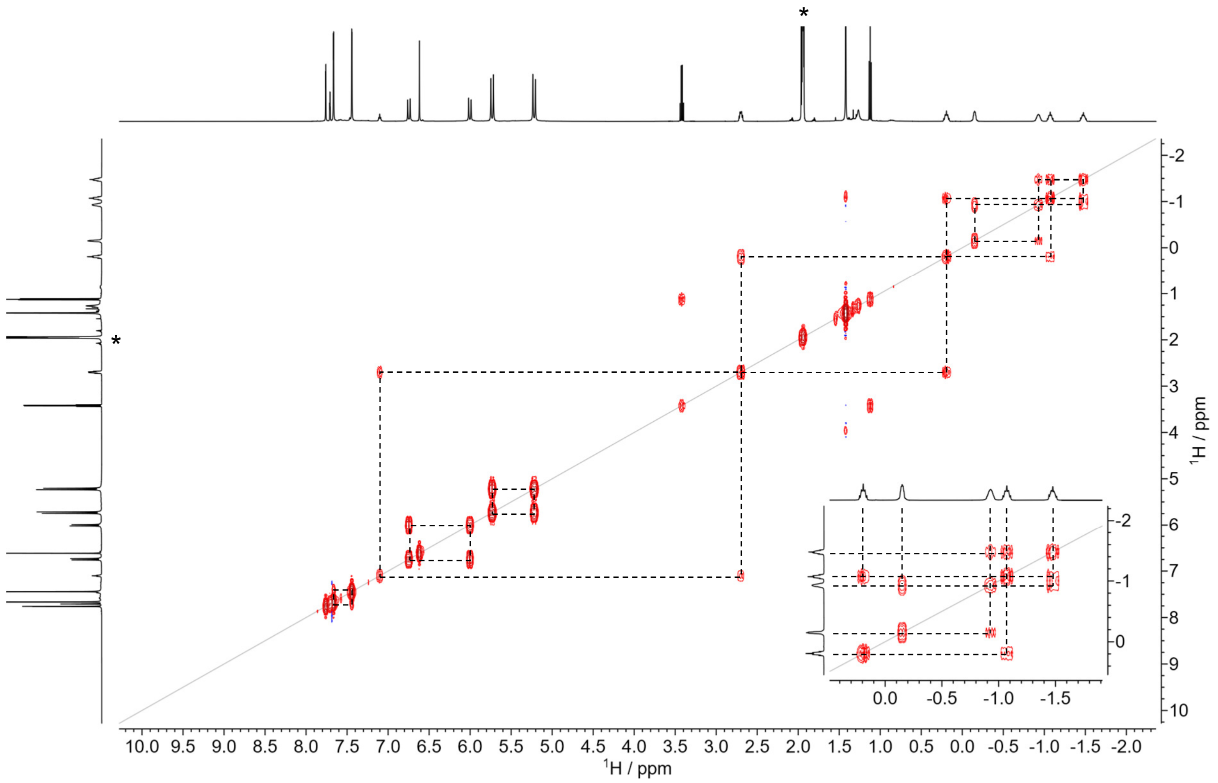

Figure S16 | ^{1}H,^{1}H COSY spectrum (500.1 MHz, 298 K) of **Rot[Cu_8L_2](OTf)$_4$** in MeCN-d_3 (* = residual solvent peaks).

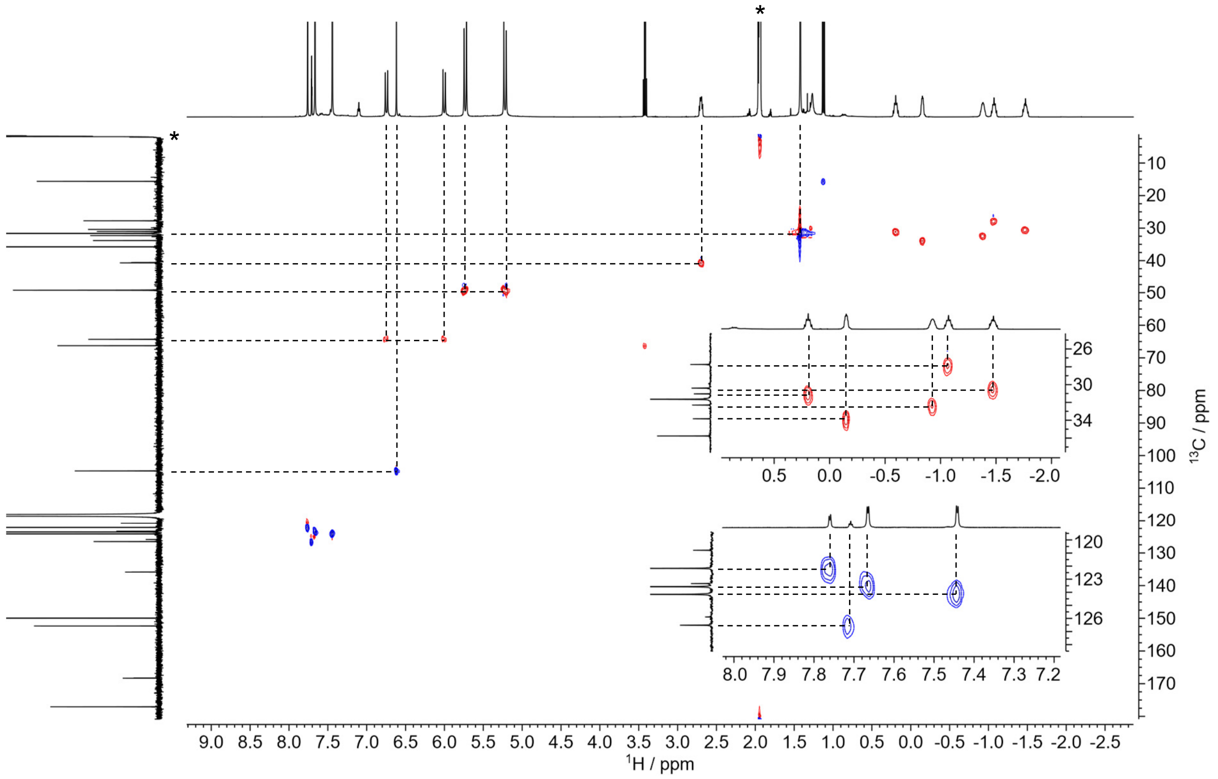

Figure S17 | ^{1}H,^{13}C HSQC spectrum (500.1 MHz, 125.8 MHz, 298 K) of **Rot[Cu_8L_2](OTf)$_4$** in MeCN-d_3 (* = residual solvent peaks).

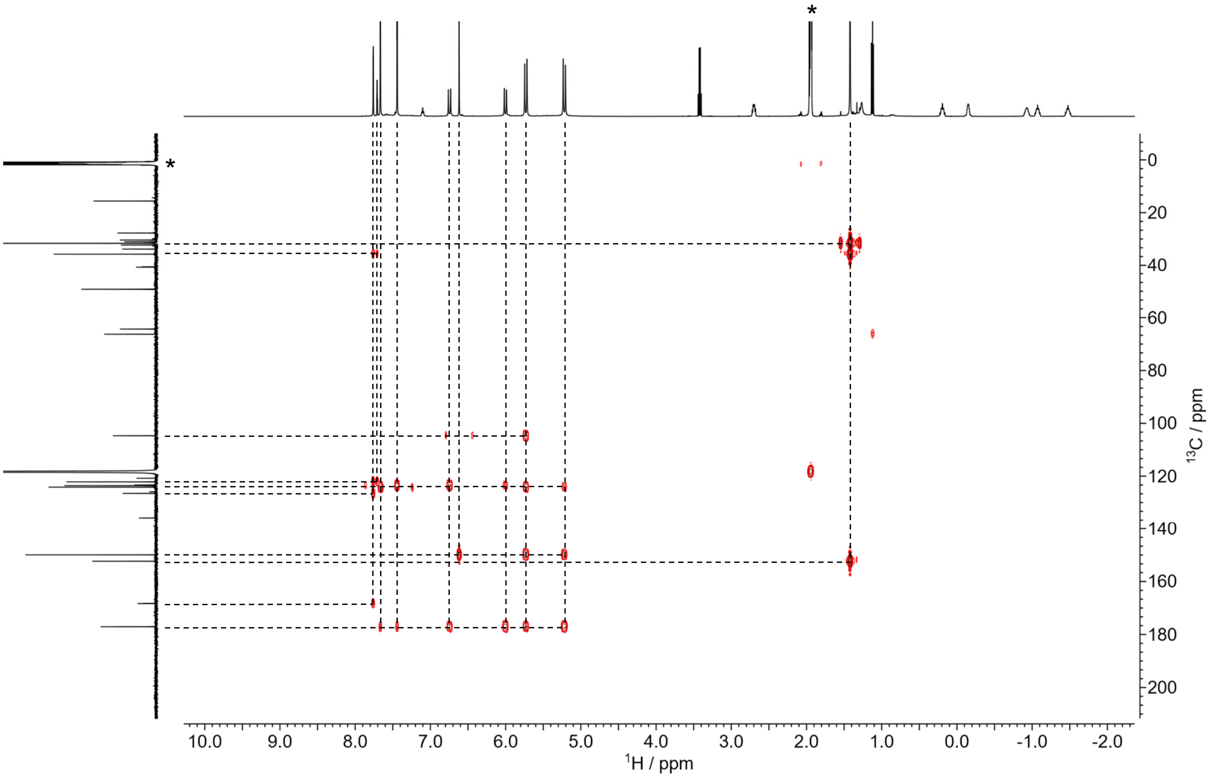

Figure S18 | ^{1}H,^{13}C HMBC spectrum (500.1 MHz, 125.8 MHz, 298 K) of **Rot[Cu$_8$L$_2$](OTf)$_4$** in MeCN-d_3 (* = residual solvent peaks).

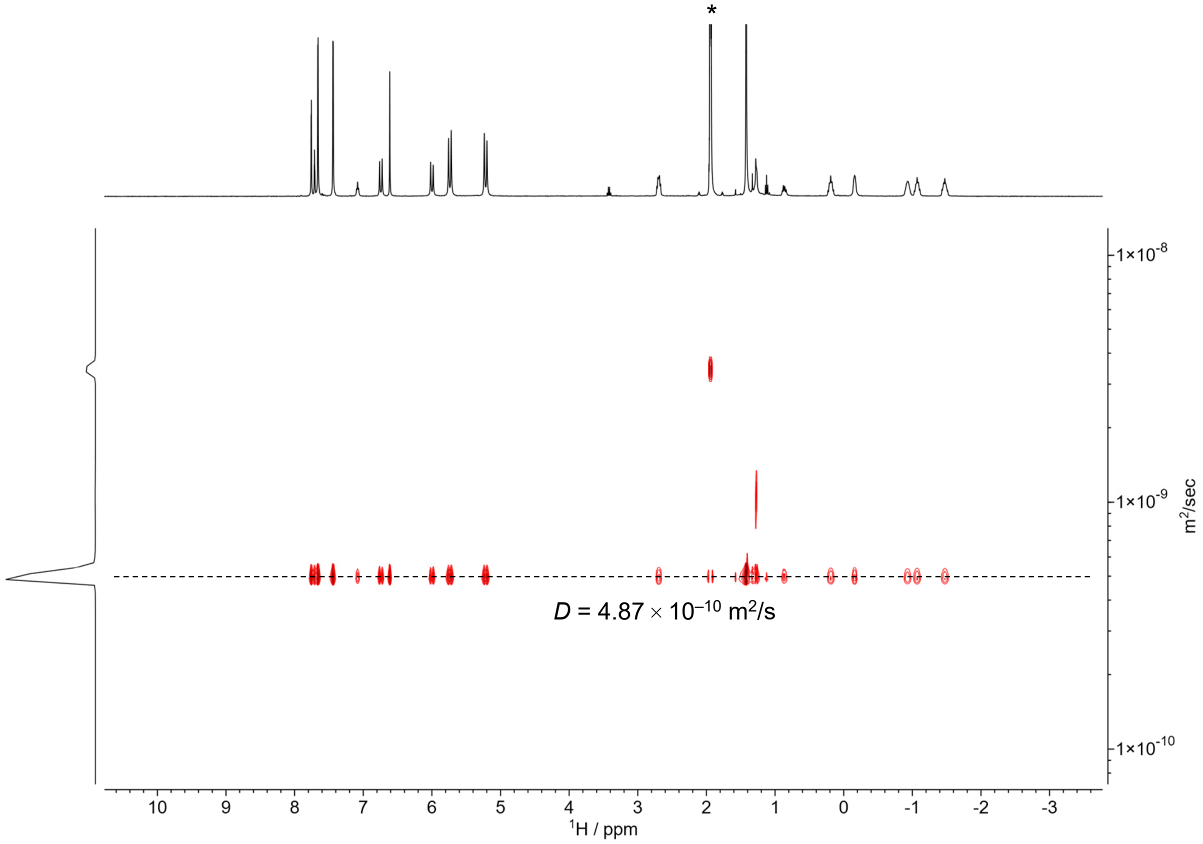

Figure S19 | ^{1}H DOSY spectrum (400.1 MHz, 298 K) of **Rot[Cu$_8$L$_2$](OTf)$_4$** in MeCN-d_3 (* = residual solvent peak).

5. Determination of Affinity Constants

Sample Preparation and Titration Procedure

To determine the affinity constant (K_a) for the interaction between **[Cu_8L_2](OTf)$_4$** (host) and tetrahydrofuran (THF, guest) in solution, a ^1H NMR titration experiment in D_2O was performed. All sample preparation steps, including weighing, addition and dilution of stock solutions, were performed under ambient conditions. **[Cu_8L_2](OTf)$_4$** (2.3 mg, 1.12 µmol) was weighed into an NMR tube using an analytical balance (precision ± 0.1 mg) and D_2O (0.789 g) was added as the solvent. Then, a benzene-d_6 capillary was added to the tube as an external standard and a ^1H NMR spectrum was recorded for reference. Aliquots of a freshly prepared stock solution of THF in D_2O (~ 55 mM) were sequentially titrated into the NMR tube containing the pillarplex solution. After each addition, ^1H NMR spectra were recorded to monitor changes in chemical shifts associated with the insertion of the guest into the pillarplex pore. The precise molar ratios of the pillarplex to THF and MeCN at each titration point were calculated by comparing the integrals of the pillarplex backbone protons with the THF- and MeCN-associated ones.

NMR Data Acquisition and Processing

^1H NMR spectra were collected using a *Bruker* AV-500 spectrometer at 20°C (±1°C). Following each guest addition, the sample was gently mixed, and a spectrum was recorded (64 scans, acquisition time = 4.0 seconds). An additional spectrum was collected with the external benzene-d_6 capillary in place. Data processing was performed in *MestReNova* 15.0.1 using the following protocol:

1. Each raw spectrum was processed *via* the "advised processing" routine as implemented in *MestReNova*, including the application of an apodization function along T_1 (Stanning 8), group delay (linear phase shift), and phase correction (regions analysis).
2. Exponential line broadening of 0.3 Hz was applied to enhance signal-to-noise ratio.
3. Then the spectra were referenced to the residual solvent signal of benzene-d_6.
4. Baseline correction ("ablative" mode with 3 points and 10 passes) was applied.

The CH_2 group directly adjacent to the oxygen in THF was identified as most sensitive to binding effects and tracked throughout the titration. Additionally, the resonance of the residual MeCN in the pillarplex pore showed significant shifts upon THF addition (Figure S20), indicating that both THF and MeCN were interacting competitively with the host in D_2O. The integrated signals of THF and MeCN were converted into cumulative molar concentrations using the initial molar ratio of pillarplex to THF and considering the exact amount of THF solution added at each titration point.

Data Fitting

Affinity constants (K_a) for THF and MeCN were determined using the *Musketeer* software package.[7] As only a single averaged signal was observed for THF and MeCN, respectively, the "fast exchange" option was selected in *Musketeer*, treating the chemical shift values as proportional to the mole fractions of the bound and free species. A competitive binding model was chosen, accounting for the simultaneous binding of both THF and MeCN to the pillarplex host, according to the following equilibrium:

$$[Cu_8L_2]^{4+} + THF + MeCN \rightleftharpoons THF \subset [Cu_8L_2]^{4+} + MeCN \rightleftharpoons MeCN \subset [Cu_8L_2]^{4+} + THF$$

Both affinity constants, K_{MeCN} and K_{THF}, were determined *via* global fitting in *Musketeer*, with the results shown in (Figure S24 and Table S1).

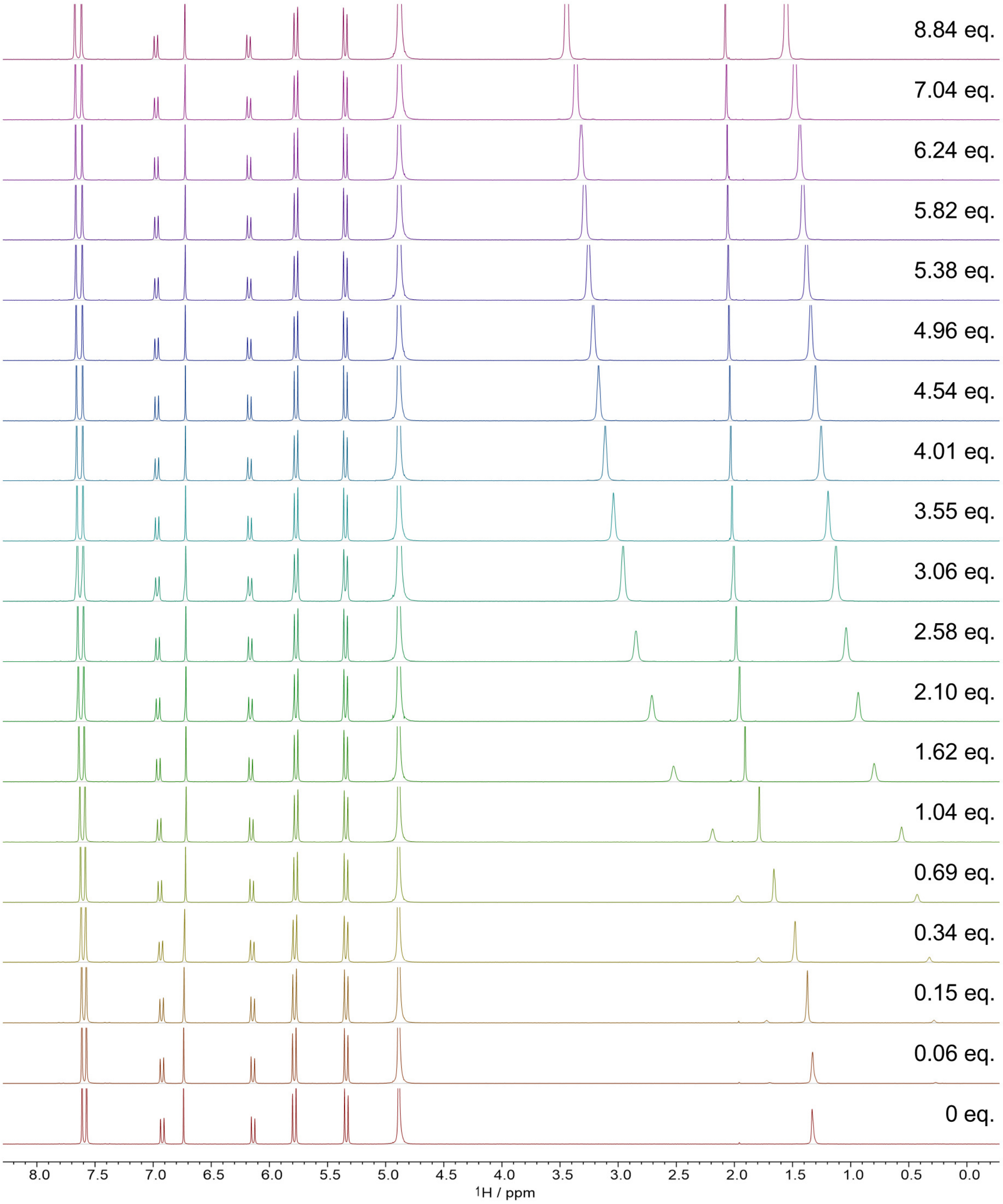

Figure S20 | ^{1}H NMR spectra (500 MHz, 294 K) of **$[Cu_8L_2](OTf)_4$** (1.57 mM in D_2O) with increasing tetrahydrofuran concentration. The singlet at 1.33 ppm in the spectrum of pure **$[Cu_8L_2](OTf)_4$** corresponds to MeCN retained within the pillarplex pore, which could not be removed by drying. The strong shift compared to unbound MeCN (δ_{free} = 2.06 ppm) indicates the binding of MeCN to **$[Cu_8L_2](OTf)_4$** in D_2O. During the titration, the MeCN signal gradually returns to 2.06 ppm.

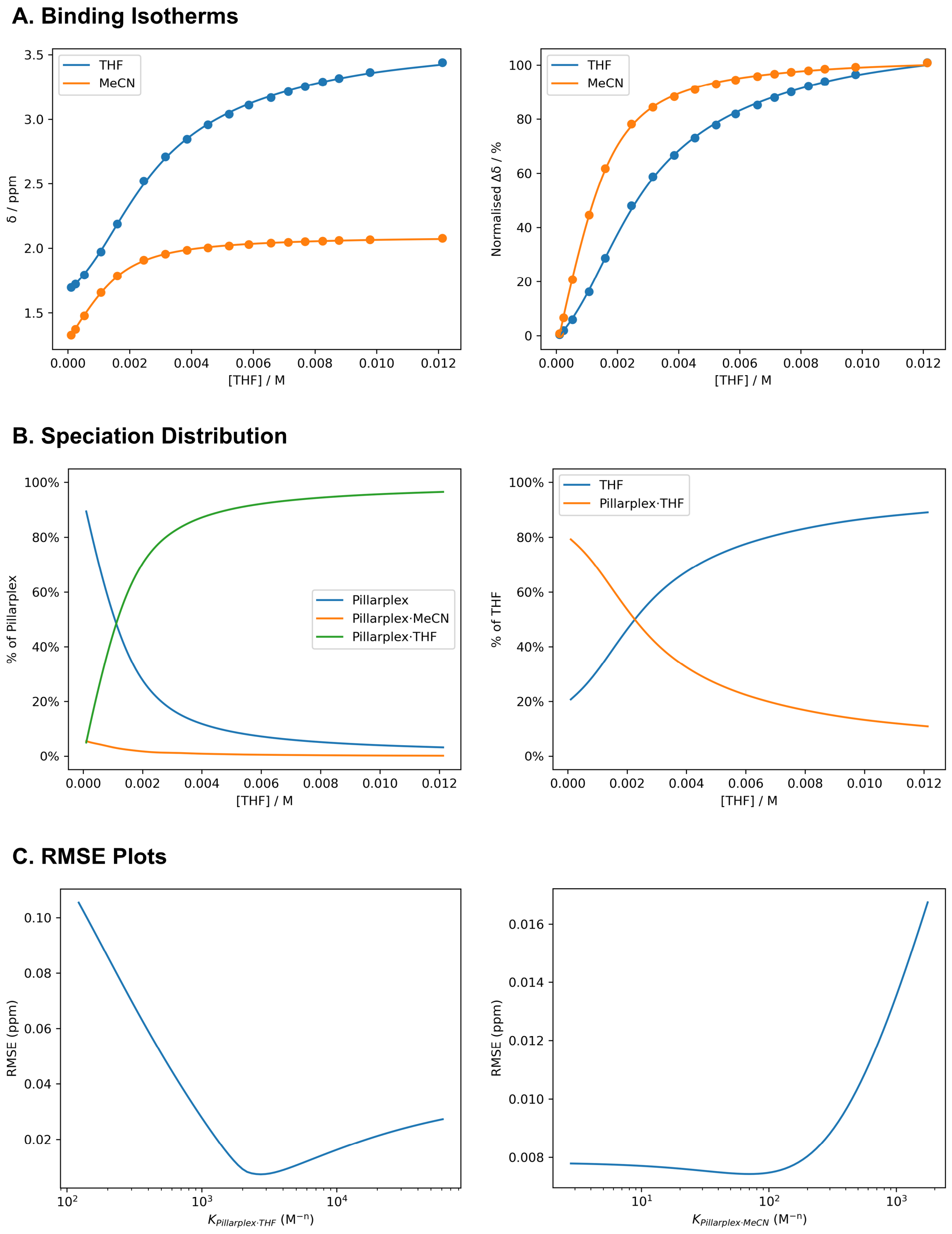

Figure S21 | Fitted ^{1}H NMR titration data for the competitive binding of THF and MeCN to **[Cu_8L_2](OTf)$_4$**: **A**. Absolute and normalised chemical shift changes for the oxygen-bound CH_2 proton of THF and the CH_3 group of MeCN, plotted against the THF concentration; **B**. Calculated distribution of different host-guest species present during the titration; **C**. Root mean square error (RMSE) plots for fitted affinity constants K_{THF} = 2730 M^{-1} and K_{MeCN} = 70 M^{-1} with an RMSE of $7.4 \cdot 10^{-3}$ ppm. The RMSE curve for K_{THF} shows a distinct minimum, enabling accurate determination of the optimal association constant. In contrast, the RMSE curve for K_{MeCN} curve lacks a well-defined minimum, indicating that any value below 10^2 M^{-1} yields a similar fit quality, thus preventing a reliable determination of K_{MeCN}.

Table S1 | Molar concentrations (mM) for pillarplex **$[Cu_8L_2](OTf)_4$** (abbreviated as 'Host'), THF and MeCN as well as experimental chemical shifts δ (ppm) of the competing guests THF and MeCN throughout the NMR titration.

[Host] / mM	[THF] / mM	[MeCN] / mM	[THF]/[Host]	δ_{THF} / ppm	δ_{MeCN} / ppm
1.565	0.099	0.970	0.06	1.697	1.328
1.557	0.238	0.958	0.15	1.724	1.372
1.552	0.528	0.968	0.34	1.793	1.478
1.539	1.066	0.942	0.69	1.972	1.657
1.527	1.595	0.951	1.04	2.188	1.786
1.514	2.454	0.964	1.62	2.523	1.909
1.503	3.154	1.142	2.10	2.710	1.956
1.492	3.849	1.164	2.58	2.847	1.985
1.481	4.532	1.184	3.06	2.958	2.005
1.470	5.217	1.160	3.55	3.041	2.020
1.459	5.857	1.163	4.01	3.112	2.031
1.448	6.571	1.145	4.54	3.169	2.041
1.438	7.128	1.142	4.96	3.216	2.048
1.428	7.676	1.122	5.38	3.254	2.053
1.417	8.242	1.104	5.82	3.290	2.057
1.406	8.772	1.079	6.24	3.319	2.062
1.389	9.779	1.048	7.04	3.364	2.067
1.371	12.124	1.008	8.84	3.442	2.079

Table S2 | Fitted chemical shift data (δ) for each species involved in the insertion equilibria between pillarplex **$[Cu_8L_2](OTf)_4$**, THF and MeCN.

Species	THF	MeCN
Free MeCN	–	2.10
MeCN in MeCN ⊂ **$[Cu_8L_2]$**	–	–6.61
Free THF	3.70	–
THF in THF ⊂ **$[Cu_8L_2]$**	1.16	–

6. HR-MS Spectra

[Cu_8L_2](OTf)$_4$

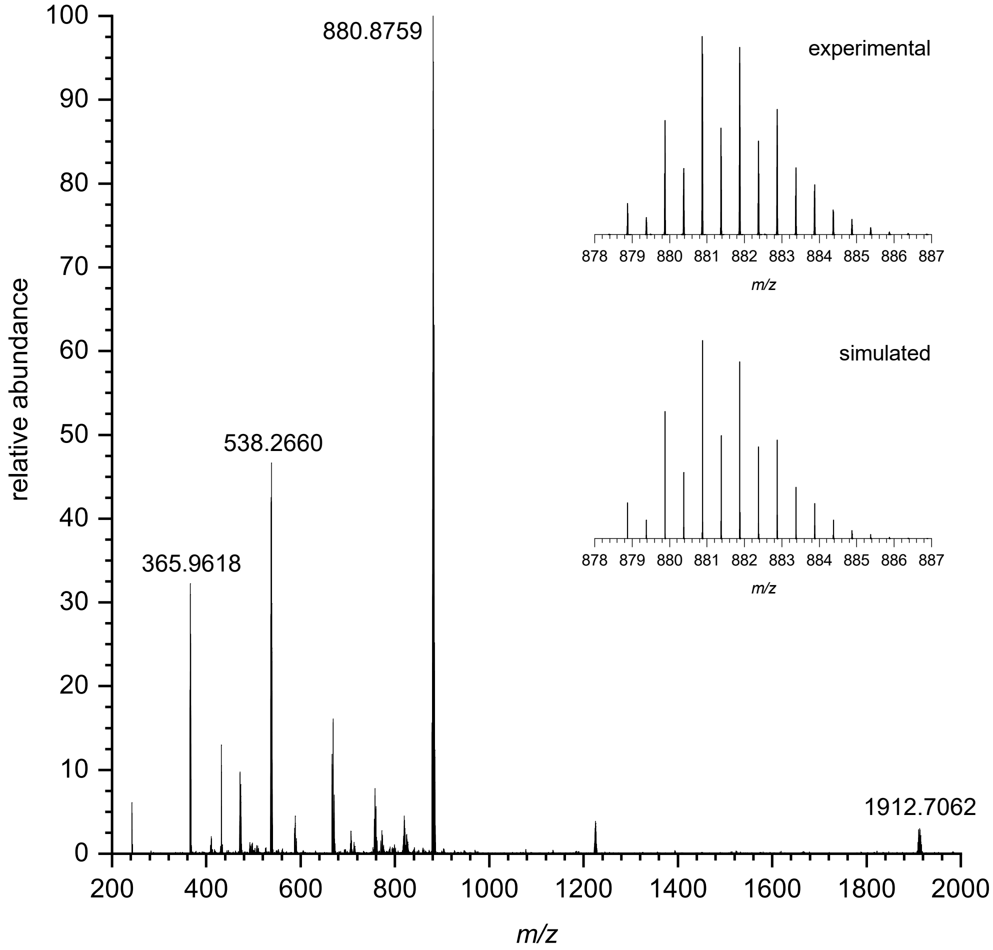

Figure S22 | HR-MS (ESI$^+$, MeCN) spectrum of **[Cu_8L_2](OTf)$_4$** and an excerpt showing **[$Cu_8L_2(OTf)_2$]$^{2+}$** (top: experimental, bottom: simulated).

Rot[Cu_8L_2](OTf)$_4$

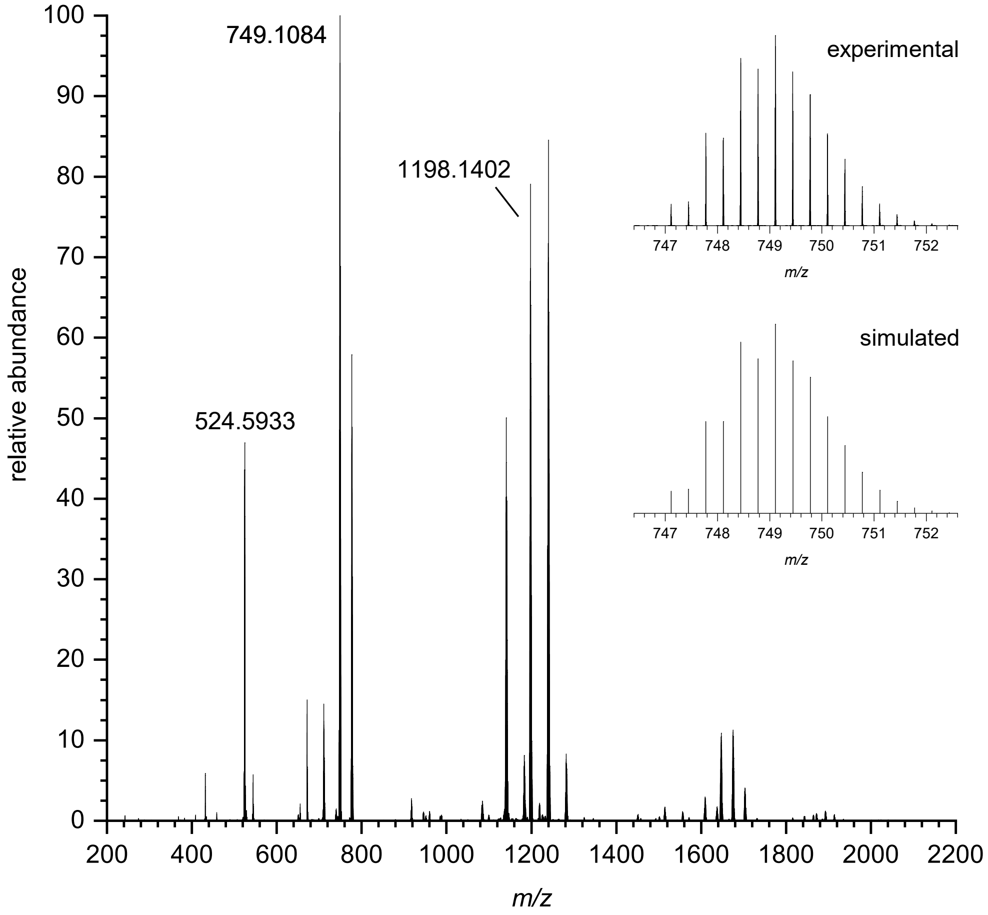

Figure S23 | HR-MS (ESI$^+$, MeCN) spectrum of **Rot[Cu_8L_2](OTf)$_4$** and an excerpt showing **Rot[Cu_8L_2(OTf)]$^{3+}$** (top: experimental, bottom: simulated).

7. Cyclic Voltammetry

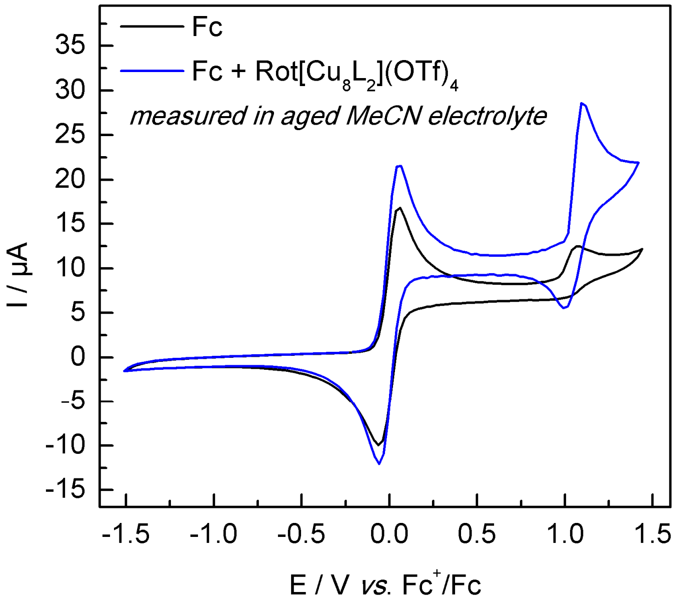

Figure S24 | Overlay plot of cyclic voltammograms measured in electrolyte solution prepared from aged MeCN with 0.1M $[Me_4N]PF_6$ and ferrocene for referencing as the blank (black trace) and after addition of **Rot[Cu_8L_2]($OTf)_4$** (blue trace). Data were measured at a scan rate of 50 mV/s, using a 2 mm GC disk working, Pt counter and Ag wire pseudo-reference electrode with ferrocene added for referencing. The blue trace clearly shows a quasi-reversible oxidation peak at +1.05 V, which according to EPR spectro-electrochemistry (*c.f.* Figure S27) is associated with the generation of Cu^{II}.

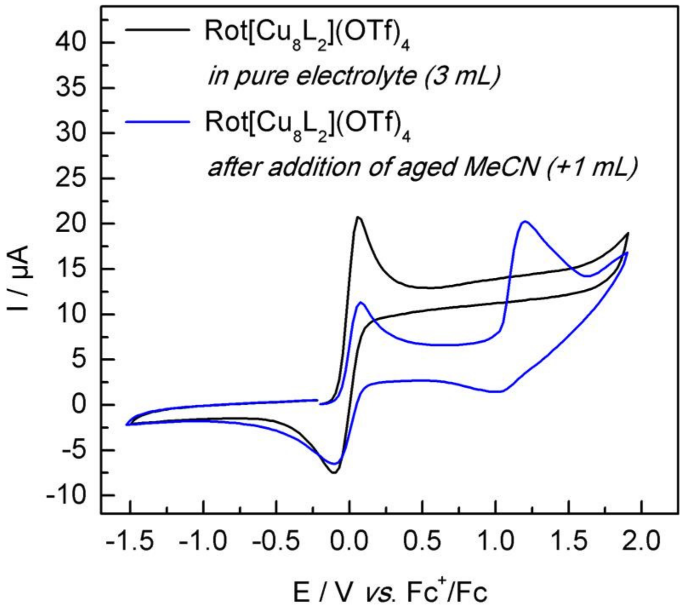

Figure S25 | Overlay plot of cyclic voltammograms measured for solutions **Rot[Cu_8L_2]($OTf)_4$** (black) in a 0.1M $[Me_4N]PF_6$ solution made from pure MeCN, and after addition of 33 vol% of aged MeCN that contains redox-triggering MeCN degradation products, upon which Cu^{I} oxidation of the rotaxane is observed (blue). Data were measured at a scan rate of 50 mV/s, using a 2 mm GC disk working, Pt counter and Ag wire pseudo-reference electrode with ferrocene added for referencing.

To further demonstrate the electrochemical inertness of the ligand at the extreme oxidation potential observed for Cu^{I} oxidation in the copper rotaxane **Rot[Cu_8L_2](OTf)$_4$**, the structurally identical Ag rotaxane **Rot[Ag_8L_2](PF_6)$_4$** bearing redox inert Ag^{I} ions instead of redox-active Cu^{I} ions was electrochemically characterised by CV. The overlay plot in Figure S32 compares CV data for the newly reported and redox reactive **Rot[Cu_8L_2](OTf)$_4$** with the other previously reported Ag^{I} rotaxane.[2] The direct comparison clearly shows that no redox activity is observed for the Ag^{I} pillarplex rotaxane up to a potential of +1.5 V vs. Fc^{+}/Fc; thus, confirming that the observed redox activity of **Rot[Cu_8L_2](OTf)$_4$** is metal-based and not ligand-based, given that the NHC ligands are basically in an identical coordination environment as in the Cu^{I} congener.

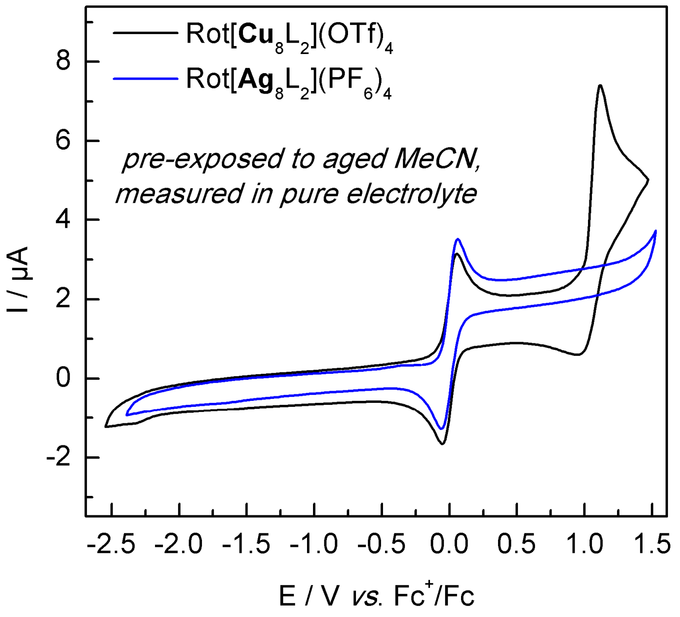

Figure S26 | Overlay plot of cyclic voltammograms measured for solutions of **Rot[Ag_8L_2](PF_6)$_4$** (blue, no redox wave except the ferrocene couple) and **Rot[Cu_8L_2](OTf)$_4$** (black, obvious Cu^{I} oxidation wave in addition to ferrocene oxidation). Both samples were pre-treated by exposure to redox-triggering MeCN degradation products, by dissolving **Rot[Cu_8L_2](OTf)$_4$** in aged MeCN, followed by removal of the solvent *in vacuo*. Data were measured in a 0.1M [Me_4N]PF_6 solution prepared from pure MeCN (scan rate: 50 mV/s, using a 2 mm GC disk working, Pt counter and Ag wire pseudo-reference electrode with ferrocene added for referencing).

8. EPR Spectroscopic Details

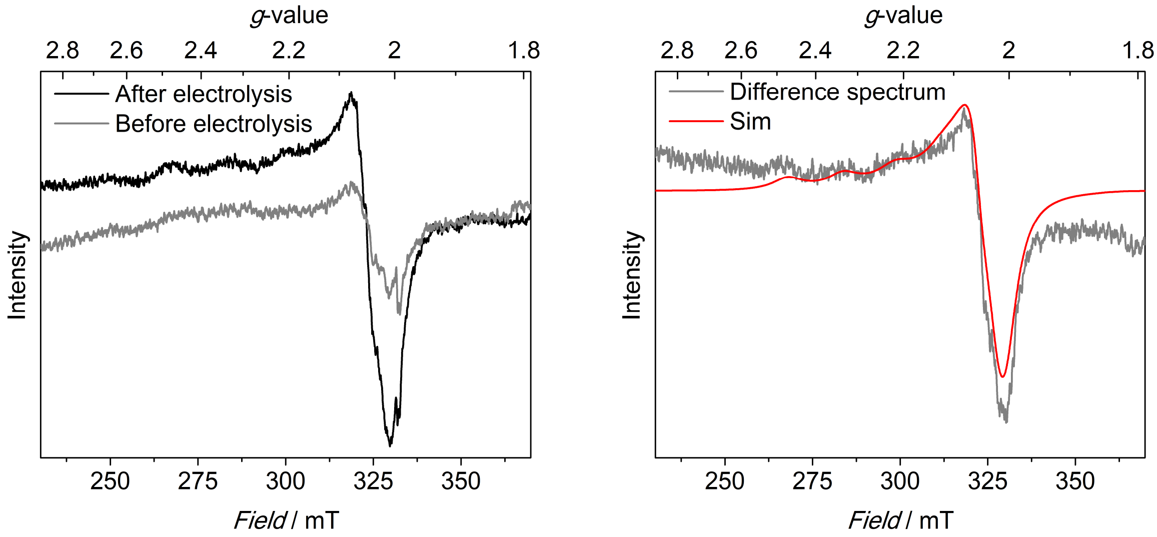

Figure S27 | Left: X-Band EPR spectrum of **Rot[Cu_8L_2](OTf)$_4$** before (grey) and after (black) electrolysis (ν = 9.267 GHz, P = 5.0 mW, modulation width = 0.4 mT, T = 133 K). The characteristic spike at g = 2.000 originates from an impurity in the EPR tube glass that was also measured in the empty EPR tube under identical conditions, and it remained unchanged upon electrolysis. After electrolysis, a weak but significantly more intense EPR signal, clearly ascribable to a Cu^{II} species was observed with resolved hyperfine coupling to the 3/2 spin Cu centre on the first g-value. **Right:** Difference spectrum (grey) obtained by subtracting the EPR signal measured before electrolysis from the EPR signal obtained after electrolysis. An overlay with the simulated EPR spectrum (red) shows great agreement with the difference spectrum. The simulated spectrum was generated with an EPR simulation program W95EPR[6] with identical parameters as presented in Figure 5 of the main text (simulation parameters: g_1 = 2.279, g_2 = 2.058, g_3 = 2.027 and line widths of W_1 = 5.5 mT, W_2 = 3.9 mT, and W_3 = 3.9 mT, coupling constant A_1 = 44 mT on g_1, and unresolved hyperfine coupling on g_2 and g_3). This further supports that the Cu^{II} species observed by EPR spectroscopy was indeed generated by electrolysis.

9. Crystallographic Details

[Cu_8L_2]($OTf)_4$

Single crystals of **[Cu_8L_2]($OTf)_4$** were grown through slow diffusion of THF into a solution of the compound in acetonitrile. A colorless, needle-shaped crystal of **[Cu_8L_2]($OTf)_4$** coated with perfluorinated ether and fixed on top of a Kapton micro sampler was used for X-ray crystallographic analysis. X-ray intensity data were collected at 100(2) K on a *Bruker* D8 VENTURE three-angle diffractometer with a TXS rotating anode with MoK_α radiation (λ = 0.71073 Å) using APEX4.[8] The diffractometer was equipped with a Helios optic monochromator, a *Bruker* PHOTON III detector, and an *Oxford* Cryostream low temperature device.

A matrix scan was used to determine the initial lattice parameters. All data were integrated with the SAINT software package using a narrow-frame algorithm, and the reflections were corrected for Lorentz and polarization effects, scan speed, and background.[8-9] The integration of the data using a monoclinic unit cell yielded a total of 20702 reflections within a 2θ range [°] of 2.83 to 50.70 (0.83 Å), of which 8513 were independent. Data were corrected for absorption effects including odd and even ordered spherical harmonics by the multi-scan method SADABS.[8,10] Space group assignment was based upon systematic absences, E statistics, and successful refinement of the structure.

The structure was solved by direct methods using SHELXT and refined by full-matrix least-squares methods against F^2 by minimizing $\Sigma w(F_o^2 - F_c^2)^2$ using SHELXL in conjunction with SHELXLE.[11-13] All non-hydrogen atoms were refined with anisotropic displacement parameters. Hydrogen atoms were refined isotropically on calculated positions using a riding model with their U_{iso} values constrained to 1.5 times the U_{eq} of their pivot atoms for terminal sp^3 carbon atoms and a C–H distance of 0.98 Å. Non-methyl hydrogen atoms were refined using a riding model with methylene, aromatic, and other C–H distances of 0.99 Å, 0.95 Å, and 1.00 Å, respectively, and U_{iso} values constrained to 1.2 times the U_{eq} of their pivot atoms.

Co-crystallised tetrahydrofuran (THF) as well as triflate counter ions were disordered and modelled using free variables in conjunction with ISOR, SIMU, RIGU, SADI, and SAME restraints as implemented in the DSR plugin in SHELXLE.[14-15] The unit cell contained additional disordered THF molecules which could not be modelled reasonably and were treated as a diffuse contribution to the overall scattering without specific atom positions using the SQUEEZE routine implemented in PLATON.[16]

Neutral atom scattering factors for all atoms and anomalous dispersion corrections for the non-hydrogen atoms were taken from International Tables for Crystallography.[17] Supplementary crystallographic data reported in this paper have been deposited with the Cambridge Crystallographic Data Centre (CCDC 2402207) and can be obtained free of charge from The Cambridge Crystallographic Data Centre via www.ccdc.cam.ac.uk/structures.[18] Images of the crystal structure were generated with *PyMOL* and *Mercury*.[19-20]

For more information on crystal data and structure refinement, see Table S2.

Rot[Cu_8L_2](OTf)$_4$

Single crystals of **Rot[Cu_8L_2](OTf)$_4$** were obtained by slow evaporation of a saturated acetonitrile solution of the compound. A colorless, needle-shaped crystal of **Rot[Cu_8L_2](OTf)$_4$** coated with perfluorinated ether and fixed on top of a Kapton micro sampler was used for X-ray crystallographic analysis. X-ray intensity data were collected at 100(2) K on a *Bruker* D8 VENTURE three-angle diffractometer with a TXS rotating anode with MoK_α radiation (λ = 0.71073 Å) using APEX4.[8] The diffractometer was equipped with a Helios optic monochromator, a *Bruker* PHOTON III detector, and an *Oxford* Cryostream low temperature device.

A matrix scan was used to determine the initial lattice parameters. All data were integrated with the SAINT software package using a narrow-frame algorithm, and the reflections were corrected for Lorentz and polarization effects, scan speed, and background.[8-9] The integration of the data using a monoclinic unit cell yielded a total of 341004 reflections within a 2θ range [°] of 3.77 to 51.24 (0.82 Å), of which 21942 were independent. Data were corrected for absorption effects including odd and even ordered spherical harmonics by the multi-scan method SADABS.[8,10] Space group assignment was based upon systematic absences, E statistics, and successful refinement of the structure.

The structure was solved by direct methods using SHELXT and refined by full-matrix least-squares methods against F^2 by minimizing $\Sigma w(F_o^2 - F_c^2)^2$ using SHELXL in conjunction with SHELXLE.[11-13] All non-hydrogen atoms were refined with anisotropic displacement parameters. Hydrogen atoms were refined isotropically on calculated positions using a riding model with their U_{iso} values constrained to 1.5 times the U_{eq} of their pivot atoms for terminal sp^3 carbon atoms and a C–H distance of 0.98 Å. Non-methyl hydrogen atoms were refined using a riding model with methylene, aromatic, and other C–H distances of 0.99 Å, 0.95 Å, and 1.00 Å, respectively, and U_{iso} values constrained to 1.2 times the U_{eq} of their pivot atoms.

Co-crystallised acetonitrile as well as triflate counter ions and some tert-butyl groups were disordered and modelled using free variables in conjunction with ISOR, SIMU, RIGU, SADI, and SAME restraints as implemented in the DSR plugin in SHELXLE.[14-15] The unit cell contained additional disordered solvent molecules which could not be modelled reasonably and were treated as a diffuse contribution to the overall scattering without specific atom positions using the SQUEEZE routine implemented in PLATON.[16]

Neutral atom scattering factors for all atoms and anomalous dispersion corrections for the non-hydrogen atoms were taken from International Tables for Crystallography.[17] Supplementary crystallographic data reported in this paper have been deposited with the Cambridge Crystallographic Data Centre (CCDC 2222645) and can be obtained free of charge from The Cambridge Crystallographic Data Centre via www.ccdc.cam.ac.uk/structures.[18] Images of the crystal structure were generated with *PyMOL* and *Mercury*.[19-20]

For more information on crystal data and structure refinement, see Table S2.

1,12-Diaminododecane

Single crystals of 1,12-diaminododecane were obtained by sublimation (85 °C, 0.050 mbar) over three hours at ambient temperature. A low-resolution dataset of the crystal structure of the compound has already been reported to the CCDC (790189) without structure factors. This redetermination provides a high-resolution dataset (0.75 Å), including structure factors. It offers slightly improved C-C bond precision, enhancing the accuracy of the original structure.

A colorless, plate-shaped crystal of 1,12-diaminododecane coated with perfluorinated ether and fixed on top of a Kapton micro sampler was used for X-ray crystallographic analysis. X-ray intensity data were collected at 100(2) K on a *Bruker* D8 VENTURE three-angle diffractometer with a TXS rotating anode with MoK_{α} radiation (λ = 0.71073 Å) using APEX4.[8] The diffractometer was equipped with a Helios optic monochromator, a *Bruker* PHOTON III detector, and an *Oxford* Cryostream low temperature device.

A matrix scan was used to determine the initial lattice parameters. All data were integrated with the SAINT software package using a narrow-frame algorithm, and the reflections were corrected for Lorentz and polarization effects, scan speed, and background.[8-9] The integration of the data using a monoclinic unit cell yielded a total of 13629 reflections within a 2θ range [°] of 5.02 to 56.61 (0.75 Å), of which 1556 were independent. Data were corrected for absorption effects including odd and even ordered spherical harmonics by the multi-scan method SADABS.[8,10] Space group assignment was based upon systematic absences, E statistics, and successful refinement of the structure.

The structure was solved by direct methods using SHELXT and refined by full-matrix least-squares methods against F^2 by minimizing $\Sigma w(F_o^2 - F_c^2)^2$ using SHELXL in conjunction with SHELXLE.[11-13] All non-hydrogen atoms were refined with anisotropic displacement parameters. Hydrogen atoms were refined isotropically on calculated positions using a riding model with their U_{iso} values constrained to 1.5 times the U_{eq} of their pivot atoms for terminal sp^3 carbon atoms and a C–H distance of 0.98 Å. Non-methyl hydrogen atoms were refined using a riding model with methylene, aromatic, and other C–H distances of 0.99 Å, 0.95 Å, and 1.00 Å, respectively, and U_{iso} values constrained to 1.2 times the U_{eq} of their pivot atoms.

Co-crystallised acetonitrile as well as triflate counter ions and some tert-butyl groups were disordered and modelled using free variables in conjunction with ISOR, SIMU, RIGU, SADI, and SAME restraints as implemented in the DSR plugin in SHELXLE.[14-15] The unit cell contained additional disordered solvent molecules which could not be modelled reasonably and were treated as a diffuse contribution to the overall scattering without specific atom positions using the SQUEEZE routine implemented in PLATON.[16]

Neutral atom scattering factors for all atoms and anomalous dispersion corrections for the non-hydrogen atoms were taken from International Tables for Crystallography.[17] Supplementary crystallographic data reported in this paper have been deposited with the Cambridge Crystallographic Data Centre (CCDC 2419509) and can be obtained free of charge from The Cambridge Crystallographic Data Centre via www.ccdc.cam.ac.uk/structures.[18] Images of the crystal structure were generated with *PyMOL* and *Mercury*.[19-20]

For more information on crystal data and structure refinement, see Table S2.

Table S3 | Crystal data and structure refinement for **$[Cu_8L_2](OTf)_4$**, **Rot$[Cu_8L_2](OTf)_4$** and diaminododecane.

**Compound**	**$[Cu_8L_2](OTf)_4$**	**Rot$[Cu_8L_2](OTf)_4$**	**Diaminododecane**
CCDC number	2402207	2222645	2419509
Empirical formula	$C_{64}H_{66}Cu_8F_{12}N_{26}O_{14}S_4$	$C_{96}H_{115}Cu_8F_{12}N_{27}O_{14}S_4$	$C_6H_{14}N$
Formula weight	2287.98	2735.70	200.36
Temperature [K]	100(2)	100(2)	100(2)
Crystal system	monoclinic	monoclinic	orthorhombic
Space group (number)	*C*2/*c* (15)	*P*2$_1$/*n* (14)	*Pbca* (61)
a [Å]	24.2574(17)	20.531(6)	6.9623(8)
b [Å]	28.7535(19)	21.705(7)	5.5494(6)
c [Å]	13.5824(8)	27.047(9)	32.489(4)
α [°]	90	90	90
β [°]	96.113(3)	104.517(8)	90
γ [°]	90	90	90
Volume [Å^3]	9419.7(11)	11668(6)	1255.3(2)
Z	4	4	4
ρ_{calc} [gcm^{-3}]	1.613	1.557	1.060
μ [mm^{-1}]	1.950	1.589	0.062
F(000)	4592	5584	456
Crystal size [mm^3]	0.065×0.081×0.721	0.065×0.115×0.214	0.062×0.274×0.496
Crystal colour	colorless	colorless	colorless
Crystal shape	needle	fragment	plate
Radiation	MoK_α (λ=0.71073 Å)	MoK_α (λ=0.71073 Å)	MoK_α (λ=0.71073 Å)
2θ range [°]	2.83 to 50.70 (0.83 Å)	3.77 to 51.24 (0.82 Å)	5.02 to 56.61 (0.75 Å)
Index ranges	−29 ≤ h ≤ 15<br>−34 ≤ k ≤ 33<br>−15 ≤ l ≤ 16	−24 ≤ h ≤ 24<br>−26 ≤ k ≤ 26<br>−32 ≤ l ≤ 32	−9 ≤ h ≤ 9<br>−7 ≤ k ≤ 7<br>−43 ≤ l ≤ 43
Reflections collected	20702	341004	13634
Independent reflections	8513<br>R_{int} = 0.0430<br>R_{sigma} = 0.0642	21942<br>R_{int} = 0.0491<br>R_{sigma} = 0.0223	1556<br>R_{int} = 0.0402<br>R_{sigma} = 0.0200
Completeness	98.7%	99.9%	99.6%
Data / Restraints / Parameters	8513 / 1043 / 765	21942 / 1289 / 1596	1556 / 0 / 70
Goodness-of-fit on F^2	1.023	1.059	1.048
Final *R* indexes [$I \geq 2\sigma(I)$]	R_1 = 0.0444<br>wR_2 = 0.1063	R_1 = 0.0450<br>wR_2 = 0.1194	R_1 = 0.0384<br>wR_2 = 0.1187
Final *R* indexes [all data]	R_1 = 0.0688<br>wR_2 = 0.1177	R_1 = 0.0647<br>wR_2 = 0.1347	R_1 = 0.0485<br>wR_2 = 0.1284
Largest peak/hole [eÅ^{-3}]	0.64/−0.45	1.20/−0.88	0.36/−0.18

Determination of Structural Parameters

Geometric parameters of **[Cu$_8$L$_2$](OTf)$_4$**, including its height (h), pore diameter (d_{pore}), inner diameter (d_{inner}), and Cu–Cu separations within short Cu$_2$ pairs ($d_{Cu–Cu}$), as well as the molecular diameters of the guests, THF (d_{THF}) and 1,12-diaminododecane (d_{alkane}), were determined using the respective SC-XRD structures and are summarised in Figure S28 and Table S4.

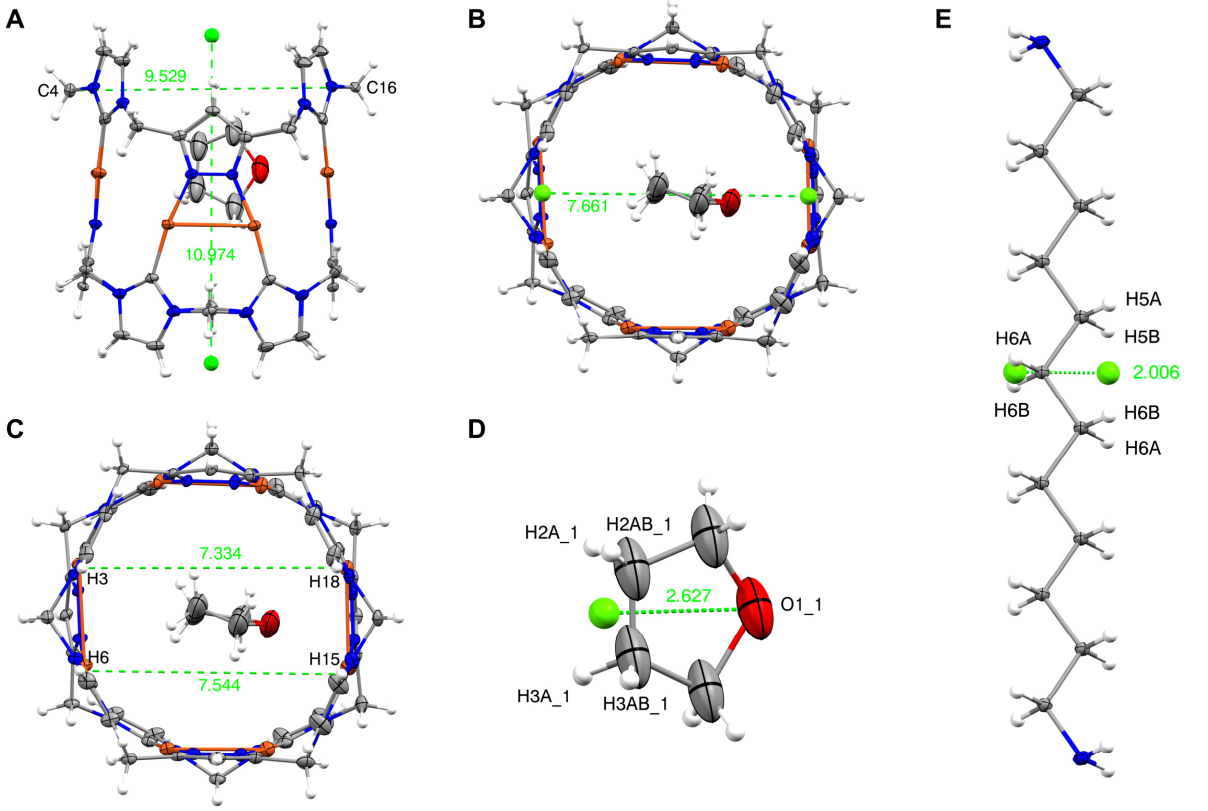

Figure S28 | Geometric parameters derived for the host and guest molecules described in this study. Ellipsoids are drawn at the 50% level of probability, solvent molecules and counterions have been removed for clarity; centroids are shown as green spheres. All distances are expressed in Å before any adjustments were made (addition or subtraction of *van der Waals* radii). **A**. Hight (h) of **[Cu$_8$L$_2$](OTf)$_4$**, defined as the distance between the centroids of all NHC backbone hydrogen atoms on the two opposing rims of the pillarplex; width (w) defined as the distance between C4 and C16; **B**. Inner diameter (d_{inner}) of **[Cu$_8$L$_2$](OTf)$_4$**, calculated as the distance between the centroids of the Cu1–Cu2 and Cu3–Cu4 pairs; **C**. Pore opening (d_{pore}) of **[Cu$_8$L$_2$](OTf)$_4$**, defined as the average distance between NHC backbone atoms H3–H18 and H6–H15; **D**. Molecular diameter (d_{THF}) of THF endohedrally coordinating to **[Cu$_8$L$_2$](OTf)$_4$**, calculated as the distance between O1_1 and the centroid of H2A_1, H2AB_1, H3A_1, and H3AB_1; **E**. Molecular diameter (d_{alkane}) of 1,12-diaminododecane, defined as the distance between the centroid of H6A and H6B and the centroid of H5A, H5B, H6A, and H6B.

Table S4 | Geometric parameters derived for the host and guest molecules described in this study, adjusted by addition or subtraction of *van der Waals* radii of hydrogen (1.10 Å), oxygen (1.52 Å), and copper (1.96 Å):[21]

**Parameter**	**Pre-adjustment / Å**	**Post-adjustment / Å**	**Comment**
Height (h)	10.974	10.974	–
Width (w)	9.529	9.529	–
Inner diameter (d_{inner})	7.661	3.741	subtracted 2 × r_{vdW}(Cu)
Pore opening (d_{pore})	7.439	5.239	subtracted 2 × r_{vdW}(H)
Molecular diameter d_{THF}	2.627	5.247	added r_{vdW}(H) + r_{vdW}(O)
Molecular diameter d_{alkane}	2.006	4.206	added 2 × r_{vdW}(H)

10. Computational Details

All density functional theory (DFT) calculations were performed using the *ORCA 6.0* quantum chemistry package.[22-23] The ωB97X-V exchange-correlation functional,[24] including non-local correlation effects,[25] was used in conjunction with the def2-TZVP triple-*ζ* valence basis set,[26] both as implemented in *ORCA*.[27-28] Tight convergence criteria were applied for self-consistent field (SCF) calculations (TightSCF) and geometry optimizations (TightOPT) in conjunction with the standard integration grid settings of *ORCA 6.0*. The RI-J approximation was employed for Coulomb integrals using the def2/J auxiliary basis set, while numerical COSX integration was used for Hartree-Fock exchange integrals.[29-32] Geometry optimizations of all compounds were based on the SC-XRD structure of **[Cu_8L_2](OTf)$_4$**. The THF conformation derived from the crystal structure was used as the starting point for the optimization of unbound THF and cyclopentane. Frequency analysis of the optimised structures confirmed convergence to an energetic minimum (except for unbound cyclopentane where a single imaginary frequency of ca. –27 cm^{-1} could not be removed despite testing rigorous grid settings and large displacements from the optimised structure).

Gaussian cube files for selected Kohn–Sham orbitals were generated using *Chemcraft* and visualised with *PyMOL*.[19,33] The electron density (ρ) and its Laplacian ($\nabla^2\rho$) of **THF ⊂ [Cu_8L_2]$^{4+}$** at bond critical points (BCPs) were computed by Quantum Theory of Atoms in Molecules (QTAIM) analysis with *Multiwfn* (Table S5 and Figure S29).[34-35]

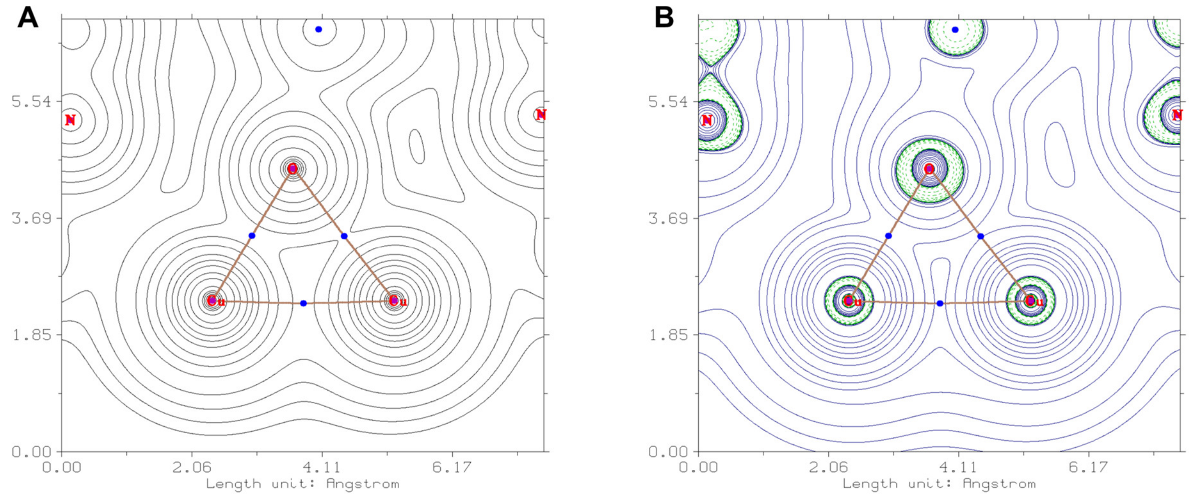

Figure S29 | QTAIM analysis of **THF ⊂ [Cu_8L_2]$^{4+}$**: Contour line plots of the electron density (**A**) and its Laplacian (**B**) along the Cu_2O_{THF} plane, showing Cu–O coordination and cuprophilic interactions within the Cu_2O_{THF} fragment. Bond paths are shown as brown lines and bond critical points (BCPs) are shown in purple (3, −3) and blue (3, −1).

The thermochemistry of the assembly of **THF ⊂ [Cu_8L_2]$^{4+}$** and **cyclopentane ⊂ [Cu_8L_2]$^{4+}$** was derived from the frequency analysis of the optimised reactants and products in the gas phase. The thermodynamic parameters ΔG^R, ΔH^R, and $T\Delta S^R$ for the following reactions were calculated based on the absolute energies listed in Table S6:

$$[Cu_8L_2]^{4+}_{(g)} + THF_{(g)} \rightleftharpoons THF \subset [Cu_8L_2]^{4+}_{(g)}$$
$$[Cu_8L_2]^{4+}_{(g)} + \text{cyclopentane}_{(g)} \rightleftharpoons \text{cyclopentane} \subset [Cu_8L_2]^{4+}_{(g)}$$

The following relations were used to calculate the thermodynamic quantities:

$$\Delta G^R = \sum G_{products} - \sum G_{reactants}$$

$$\Delta H^R = \sum H_{products} - \sum H_{reactants}$$

$$T\Delta S^R = \sum TS_{products} - \sum TS_{reactants}$$

Table S5 | Bond critical points (BCPs) between a pair of atoms (element type + atom number shown) and their associated electron density (ρ) and its Laplacian ($\nabla^2\rho$).

BCP	Distance / Å	Electron density (ρ) / a.u.	Laplacian of ρ ($\nabla^2\rho$) / a.u.
Cu3–O125	2.631	0.0204	0.0764
Cu4–O125	2.449	0.0285	0.1237
Cu1–Cu2	2.938	0.0168	0.0313
Cu3–Cu4	2.874	0.0172	0.0346
Cu63–Cu64	2.939	0.0163	0.0308
Cu65–Cu66	2.919	0.0164	0.0309

Table S6 | Thermochemical quantities (Gibbs free energy G, enthalpy H, and temperature-weighted entropy term TS) obtained by frequency analysis of the optimised structures of THF, cyclopentane, **$[Cu_8L_2]^{4+}$**, **THF ⊂ $[Cu_8L_2]^{4+}$**, and **cyclopentane ⊂ $[Cu_8L_2]^{4+}$**.

Species	G / kJ mol^{-1}	H / kJ mol^{-1}	TS / kJ mol^{-1}
THF	–610120	–610032	88
cyclopentane	–515801	–515716	85
**$[Cu_8L_2]^{4+}$**	–42772909	–42772497	411
**THF ⊂ $[Cu_8L_2]^{4+}$**	–43383070	–43382630	440
**cyclopentane ⊂ $[Cu_8L_2]^{4+}$**	–43288713	–43288270	444

Table S7 | Cartesian coordinates of the optimised structure of THF.

Atom #	Atom	x	y	z
1	O	10.3	6.896166445	9
2	C	10.81921388	7.413442111	7.839435067
3	C	11.25073993	8.851517667	8.121827773
4	C	11.64692248	8.770350865	9.597130815
5	C	10.5990683	7.800341651	10.13574729
6	H	11.67166173	6.800192013	7.516909868
7	H	10.03628013	7.342742377	7.077948319
8	H	10.40564173	9.535956104	7.994260915
9	H	12.06136825	9.182029903	7.468600601
10	H	11.63062229	9.73613074	10.10723178
11	H	12.64974516	8.342785356	9.698981891
12	H	9.680460733	8.332741024	10.42026485
13	H	10.94460276	7.223229716	10.99833043

Table S8 | Cartesian coordinates of the optimised structure of cyclopentane.

Atom #	Atom	x	y	z
1	C	10.3639611	7.17124149	9.07518153
2	C	11.0728971	7.45257036	7.74564542
3	C	11.4610967	8.94732096	7.81548056
4	C	11.3915372	9.33571835	9.31577674
5	C	11.1615304	8.01666776	10.0706035
6	H	11.9746467	6.83329395	7.68063774
7	H	10.4568925	7.21761674	6.87342659
8	H	10.7678893	9.5570727	7.22901576
9	H	12.4574926	9.11700716	7.3987193
10	H	10.5466783	10.010008	9.48998171
11	H	12.2900554	9.85807506	9.65470294
12	H	10.6525893	8.15861696	11.0281383
13	H	12.1209365	7.526511	10.2767239
14	H	10.337081	6.1080924	9.33061881
15	H	9.32763685	7.52959408	9.03309321

Table S9 | Cartesian coordinates of the optimised structure of **$[Cu_8L_2]^{4+}$**.

Atom #	Atom	x	y	z
1	Cu	12.614	11.8	9.460544226
2	Cu	14.67006287	9.757299326	9.561922875
3	Cu	7.477477515	6.669109798	9.20296613
4	Cu	9.531583221	4.610058406	9.304130279
5	N	11.28536928	12.60514047	6.937774563
6	N	13.32175632	12.04965699	6.62635646
7	N	15.04448829	10.32338318	6.710849001
8	N	15.57915212	8.304789282	7.148500059
9	N	14.19327429	6.056846521	9.326665521
10	N	13.23586033	5.097841046	9.278591729
11	N	11.1039823	3.823023406	6.923478395
12	N	9.10890373	4.380626382	6.412719486
13	N	7.384939244	6.105860434	6.328363863
14	N	6.808734687	8.123759139	6.713465852
15	N	7.971175371	10.37026506	9.020639287
16	N	8.928945433	11.32889725	9.068585588
17	C	12.38375271	12.19385223	7.59537938
18	C	11.52046084	12.71756229	5.578076476
19	C	12.80689323	12.3693054	5.379252674
20	C	14.71620961	11.72859312	6.877598019
21	C	15.13991694	9.434437581	7.730739462
22	C	15.42187424	9.747505955	5.50736761
23	C	15.75688485	8.472343136	5.785771847
24	C	15.91151442	7.07563311	7.875238784
25	C	14.74795381	6.161481712	8.110703903
26	C	14.13664003	5.258960222	7.245723925
27	C	13.19390501	4.604772719	8.03269508
28	C	12.29590442	3.454361274	7.693422081
29	C	9.945830396	4.233250642	7.469827109
30	C	11.00522113	3.714343132	5.546837014
31	C	9.74504845	4.06400249	5.222132142
32	C	7.696424776	4.700609808	6.525374293
33	C	7.189807995	6.993827181	7.334874246

34	C	7.126401283	6.682618248	5.094283993
35	C	6.765028233	7.957334216	5.339762448
36	C	6.406027252	9.352264583	7.404905011
37	C	7.540659855	10.2659303	7.755403479
38	C	8.235541404	11.16818136	6.955890991
39	C	9.095304044	11.82211182	7.833177149
40	C	10.02259654	12.97273083	7.585754446
41	H	10.77037413	13.06404074	4.883632221
42	H	13.39956628	12.35583463	4.477237667
43	H	14.95487602	12.0168976	7.900811137
44	H	15.33584085	12.3135822	6.195513936
45	H	15.45298462	10.29503073	4.577669963
46	H	16.13410388	7.68873607	5.146565467
47	H	16.68586468	6.559685063	7.303467454
48	H	16.35267501	7.366030413	8.831428147
49	H	14.37637948	5.071445673	6.208606219
50	H	12.84190795	2.709760359	7.110178691
51	H	11.9588484	2.965908296	8.610467043
52	H	11.82064761	3.369392471	4.929586314
53	H	9.244870201	4.080421246	4.265766438
54	H	7.358495384	4.411725541	7.52004143
55	H	7.147345579	4.115718488	5.78523997
56	H	7.186609438	6.136047978	4.165463098
57	H	6.451532901	8.741365156	4.667499471
58	H	5.872084862	9.061391666	8.312408596
59	H	5.692419531	9.868756191	6.759383175
60	H	8.100477694	11.35586995	5.900057384
61	H	9.537316529	13.71736157	6.95112645
62	H	10.26614192	13.46135061	8.531931205
63	Cu	9.455995001	11.81217989	10.83728049
64	Cu	7.39888748	9.754776611	10.73341697
65	Cu	14.59143654	6.670657462	11.08855617
66	Cu	12.53586074	4.610505669	10.98529711
67	N	10.78400314	12.60117041	13.36143531
68	N	8.747790993	12.04433552	13.6716722
69	N	7.02505283	10.31803319	13.58509247
70	N	6.489657066	8.300049146	13.14542813
71	N	7.874411993	6.053533799	10.96459149
72	N	8.831860048	5.094502082	11.0116847
73	N	10.96484633	3.819099352	13.36568333
74	N	12.95984567	4.377377921	13.87612012
75	N	14.68393012	6.102553324	13.96219135
76	N	15.26103251	8.120816996	13.58027338
77	N	14.09864119	10.36985577	11.27603605
78	N	13.14066599	11.32836492	11.22920965
79	C	9.685930547	12.19030892	12.70304616
80	C	10.54858807	12.71163033	14.72123651
81	C	9.262279466	12.36248635	14.91931557
82	C	7.353436509	11.72339144	13.41966568
83	C	6.929161978	9.430155777	12.56430217
84	C	6.647672055	9.741005217	14.78803492
85	C	6.31216855	8.466253189	14.50835055
86	C	6.157019819	7.071744055	12.41739385
87	C	7.320428637	6.157664144	12.18089835
88	C	7.932199232	5.254732745	13.04513062
89	C	8.874559096	4.600950268	12.25734293

90	C	9.772874715	3.450525453	12.59574617
91	C	12.12244284	4.230729751	12.81929065
92	C	11.06434867	3.708863848	14.7421429
93	C	12.32448913	4.058883401	15.06663654
94	C	14.37224687	4.697639783	13.76303331
95	C	14.87935115	6.992073861	12.95709488
96	C	14.94291935	6.677224255	15.19714884
97	C	15.30484337	7.952173551	14.95370425
98	C	15.66397493	9.35027878	12.89064801
99	C	14.52938375	10.26432384	12.54108883
100	C	13.83445718	11.16562149	13.34160927
101	C	12.97438811	11.8202744	12.46514799
102	C	12.04669421	12.97023296	12.71411365
103	H	11.298381	13.05746402	15.4163154
104	H	8.669435614	12.34749247	15.82119056
105	H	7.115172122	12.0125117	12.39659048
106	H	6.733518221	12.30780385	14.10198896
107	H	6.616893335	10.28755722	15.71831503
108	H	5.934799471	7.68211288	15.14681387
109	H	5.382593523	6.555314879	12.98863587
110	H	5.71590839	7.363224235	11.46151635
111	H	7.69301603	5.066734559	14.08228826
112	H	9.227154023	2.705480382	13.17868935
113	H	10.1098569	2.962661044	11.67836544
114	H	10.2494531	3.362671809	15.35940441
115	H	12.82508537	4.074386031	16.02279772
116	H	14.70945934	4.410417321	12.76764446
117	H	14.92185861	4.111514575	14.50179266
118	H	14.88254271	6.129237192	16.12512278
119	H	15.6187406	8.734973458	15.62721644
120	H	16.19823343	9.060704101	11.98291491
121	H	16.37731988	9.86593206	13.53712491
122	H	13.96962407	11.35221865	14.39762493
123	H	12.53165033	13.7140995	13.34987793
124	H	11.80305699	13.4601397	11.76862885

Table S10 | Cartesian coordinates of the optimised structure of **THF ⊂ $[Cu_8L_2]^{4+}$**.

Atom #	Atom	x	y	z
1	Cu	12.7569506	12.0226544	9.45611109
2	Cu	14.7830859	9.9227685	9.54081979
3	Cu	7.55819117	6.66624022	9.19569065
4	Cu	9.58583866	4.63272693	9.3070881
5	N	11.2975324	12.6673638	6.96145901
6	N	13.3202384	12.1066028	6.57984504
7	N	15.0026127	10.3495263	6.65167066
8	N	15.4846916	8.32917583	7.14388442
9	N	14.1225969	6.05786617	9.33040588
10	N	13.1981205	5.06643094	9.28160311
11	N	11.1200903	3.74279897	6.92665373
12	N	9.13221884	4.32593249	6.42164583
13	N	7.45318098	6.09558697	6.32820038
14	N	6.90818339	8.12619869	6.69698919
15	N	8.01046259	10.3914168	9.02861208
16	N	8.9460217	11.3714945	9.09821485

17	C	12.4328052	12.3060223	7.58634597
18	C	11.4612048	12.6956761	5.58708727
19	C	12.7390152	12.3441532	5.34364504
20	C	14.7200174	11.7702177	6.77262058
21	C	15.1379851	9.50316753	7.70320052
22	C	15.2653327	9.70521179	5.45238008
23	C	15.5675478	8.42982309	5.76533677
24	C	15.8286714	7.11841609	7.89658877
25	C	14.687489	6.17100379	8.11875354
26	C	14.1162607	5.23877002	7.25715183
27	C	13.1867125	4.55883352	8.04115648
28	C	12.314935	3.38800535	7.69525796
29	C	9.96482536	4.15592989	7.48002574
30	C	11.0225773	3.65081127	5.54714489
31	C	9.76639533	4.01612306	5.22632575
32	C	7.72897598	4.68186257	6.53552895
33	C	7.24337365	6.98647234	7.33000372
34	C	7.24652449	6.67921138	5.08616984
35	C	6.90475526	7.96120007	5.32081676
36	C	6.48918709	9.35231846	7.38123223
37	C	7.60584778	10.2820204	7.75479023
38	C	8.29546825	11.2055226	6.97264521
39	C	9.12401764	11.8753533	7.86854702
40	C	10.0516359	13.0313856	7.64254481
41	H	10.6740227	12.9968341	4.91275029
42	H	13.2858093	12.2823546	4.41498014
43	H	15.0193104	12.0965958	7.76819689
44	H	15.3148458	12.3089706	6.03271906
45	H	15.2527309	10.2099554	4.49827556
46	H	15.8674758	7.60428052	5.13799535
47	H	16.6342505	6.61642358	7.35565912
48	H	16.2335113	7.43824086	8.85944308
49	H	14.3740458	5.04557247	6.22525045
50	H	12.8793137	2.65866471	7.11012658
51	H	11.9841519	2.89039529	8.60973666
52	H	11.8337852	3.30154247	4.92664134
53	H	9.2658631	4.0430759	4.27035471
54	H	7.3893288	4.4130048	7.53552437
55	H	7.16149355	4.10626766	5.8017131
56	H	7.32199892	6.13186581	4.15888701
57	H	6.62620047	8.75041343	4.63920546
58	H	5.94448071	9.05495035	8.28015797
59	H	5.78129425	9.86551787	6.72650587
60	H	8.1725762	11.3960294	5.91569238
61	H	9.5616524	13.8002707	7.04076347
62	H	10.3185872	13.4856133	8.59940873
63	Cu	9.48278975	11.8342269	10.8678468
64	Cu	7.42224799	9.7433007	10.728728
65	Cu	14.5200956	6.6807799	11.089621
66	Cu	12.4671115	4.58165794	10.9800545
67	N	10.8229772	12.6135198	13.3961984
68	N	8.79886643	12.0073685	13.701834
69	N	7.07608478	10.2755583	13.5930361
70	N	6.50411347	8.27211612	13.1290766
71	N	7.87909099	6.01964247	10.9716898
72	N	8.82106764	5.0491901	11.028322

73	N	10.9155075	3.78212654	13.3733634
74	N	12.8963903	4.39583731	13.8769103
75	N	14.6060998	6.14218073	13.9585713
76	N	15.2143022	8.15182855	13.5708499
77	N	14.2322044	10.5499879	11.2571185
78	N	13.2871213	11.521055	11.2218791
79	C	9.73211239	12.188081	12.734438
80	C	10.5870494	12.6987006	14.757876
81	C	9.30885862	12.3186662	14.9528645
82	C	7.40529358	11.6816746	13.4433974
83	C	6.95820044	9.40219574	12.5603153
84	C	6.69707305	9.68782266	14.7903163
85	C	6.33976874	8.42255468	14.494903
86	C	6.14912146	7.05354174	12.3912503
87	C	7.29346581	6.11292762	12.1740248
88	C	7.8693175	5.18967929	13.0415384
89	C	8.82440281	4.53742827	12.2657511
90	C	9.72252236	3.39134466	12.6119847
91	C	12.060915	4.21781069	12.8215332
92	C	11.0188145	3.68804585	14.749992
93	C	12.270181	4.0717302	15.0707387
94	C	14.3031016	4.73350048	13.759971
95	C	14.8158388	7.02610887	12.9514788
96	C	14.8720664	6.71699019	15.1918041
97	C	15.2535746	7.98567512	14.9450161
98	C	15.6602768	9.36657255	12.8811203
99	C	14.5628799	10.3271985	12.5363918
100	C	13.8150406	11.165267	13.3586684
101	C	13.02722	11.9033165	12.4801027
102	C	12.0749511	13.0273569	12.754372
103	H	11.3300355	13.0517145	15.4567579
104	H	8.71840084	12.2780813	15.8555279
105	H	7.16932043	11.9811274	12.4227366
106	H	6.78285839	12.2592491	14.1293235
107	H	6.67976618	10.2219629	15.7281169
108	H	5.95497532	7.63517013	15.1248922
109	H	5.3495666	6.56007115	12.9478996
110	H	5.73637506	7.35986041	11.4274228
111	H	7.60299891	4.99036148	14.0700193
112	H	9.18600177	2.65047219	13.2082258
113	H	10.0586006	2.89723716	11.6976683
114	H	10.2123433	3.32856457	15.3707284
115	H	12.7710932	4.10683377	16.0262027
116	H	14.6408053	4.45033778	12.763559
117	H	14.8625157	4.15342783	14.496235
118	H	14.8025274	6.17302363	16.1215234
119	H	15.5798884	8.76505356	15.6167069
120	H	16.1784788	9.05946199	11.969794
121	H	16.3976862	9.85083703	13.5254864
122	H	13.8685866	11.2565714	14.4343078
123	H	12.5419047	13.7697872	13.4055117
124	H	11.8197402	13.5307048	11.8190013
125	O	10.1635996	6.9978446	9.0403307
126	C	10.8126676	7.42355749	7.82956088
127	C	11.2564674	8.85199812	8.10125453
128	C	11.6225688	8.7843847	9.58595626

129	C	10.6074733	7.79510629	10.1509772
130	H	11.6685802	6.76732255	7.62686352
131	H	10.0919201	7.33292462	7.01143703
132	H	10.4260942	9.54390311	7.93558804
133	H	12.0937217	9.15287521	7.46401237
134	H	11.565611	9.74925322	10.0961816
135	H	12.6378996	8.39082599	9.706835
136	H	9.73266769	8.3016057	10.5785009
137	H	11.0294633	7.13602438	10.9193245

Table S11 | Cartesian coordinates of the optimised structure of **cyclopentane** ⊂ **$[Cu_8L_2]^{4+}$**.

Atom #	Atom	x	y	z
1	Cu	12.8412302	12.097047	9.44689669
2	Cu	14.8812996	9.9638237	9.54001731
3	Cu	7.20809515	6.40373225	9.15852173
4	Cu	9.24981925	4.26534955	9.26020487
5	N	11.2643589	12.5791747	6.97573504
6	N	13.2844277	12.0332907	6.55405707
7	N	14.9725186	10.2843077	6.63141987
8	N	15.4552812	8.27383162	7.16604177
9	N	14.1260284	6.01592435	9.38182126
10	N	13.1861307	5.04081677	9.33945141
11	N	11.0641619	3.79696874	6.95460417
12	N	9.09512227	4.3430912	6.3353875
13	N	7.39674761	6.08212557	6.25049977
14	N	6.86686677	8.09173184	6.74190427
15	N	7.98762852	10.3462961	9.08416264
16	N	8.92245794	11.3258445	9.13681133
17	C	12.4351987	12.2968002	7.57903713
18	C	11.3720503	12.4966963	5.59771418
19	C	12.6468281	12.1544625	5.32885645
20	C	14.6919579	11.7093472	6.71196472
21	C	15.1585707	9.47305208	7.70314909
22	C	15.1577851	9.59501813	5.4426219
23	C	15.4600768	8.32694174	5.78214614
24	C	15.8178965	7.07625582	7.93256675
25	C	14.6866919	6.11784505	8.16743898
26	C	14.0965922	5.19069944	7.31151934
27	C	13.1516645	4.53508162	8.09905759
28	C	12.2226202	3.40291886	7.76464596
29	C	9.83596063	4.06773448	7.43823818
30	C	11.0966908	3.89442937	5.57343741
31	C	9.85481819	4.23665391	5.18001111
32	C	7.67648049	4.65820855	6.35279521
33	C	7.10522751	6.88976345	7.30122706
34	C	7.33148561	6.77624639	5.05172223
35	C	6.99913542	8.04372129	5.36411881
36	C	6.44356535	9.28963946	7.4752353
37	C	7.55337768	10.2409596	7.81938951
38	C	8.22333413	11.1701692	7.02674144
39	C	9.0787962	11.8318938	7.90612985
40	C	10.030499	12.9685943	7.66719592
41	H	10.5518281	12.7266819	4.93499565
42	H	13.1561271	12.0296027	4.38543306

43	H	15.0224053	12.0724774	7.6847948
44	H	15.2602787	12.2214242	5.93305003
45	H	15.0989172	10.0671124	4.47374651
46	H	15.7143893	7.47683003	5.16775107
47	H	16.6303638	6.57892417	7.39733854
48	H	16.2174684	7.41413051	8.89133153
49	H	14.3428184	4.99700329	6.27697906
50	H	12.7558947	2.62134696	7.21809152
51	H	11.8396061	2.9559517	8.68486006
52	H	11.9808902	3.67440223	4.99496853
53	H	9.44374511	4.37126022	4.19113571
54	H	7.25242524	4.28814312	7.28590614
55	H	7.19242252	4.14687491	5.51840777
56	H	7.48485531	6.30737159	4.09167929
57	H	6.8086017	8.89656146	4.73070471
58	H	5.95376118	8.95211131	8.39129592
59	H	5.68831096	9.79496436	6.86844309
60	H	8.08109568	11.3634362	5.97278203
61	H	9.55150581	13.7486594	7.07059514
62	H	10.3181586	13.4157374	8.62140219
63	Cu	9.48557844	11.7941891	10.9002057
64	Cu	7.43847015	9.693849	10.7911834
65	Cu	14.5002542	6.67328802	11.1338325
66	Cu	12.4538948	4.57389542	11.0393913
67	N	10.8708927	12.5806579	13.4035328
68	N	8.87045839	11.9200711	13.7534206
69	N	7.14621369	10.1789349	13.6630633
70	N	6.51839412	8.19687268	13.1753142
71	N	7.6053909	5.76352616	10.9145904
72	N	8.54560133	4.78821745	10.9632823
73	N	10.8338333	3.78670468	13.3939215
74	N	12.7895483	4.44809791	13.9397508
75	N	14.5136729	6.19006936	14.0195158
76	N	15.1869645	8.17237251	13.5972718
77	N	14.3336843	10.6180957	11.2506976
78	N	13.393038	11.5926387	11.2101123
79	C	9.77381494	12.1377214	12.7640569
80	C	10.668949	12.6402027	14.7720827
81	C	9.40603216	12.2266806	14.9951771
82	C	7.47390332	11.5883939	13.5246307
83	C	6.99117112	9.32595983	12.6187259
84	C	6.77180047	9.57982132	14.8564665
85	C	6.37889591	8.3286876	14.5466562
86	C	6.10293417	7.00975722	12.4209142
87	C	7.20294155	6.0250577	12.1659926
88	C	7.89613168	5.20954605	13.0549053
89	C	8.73085156	4.44520655	12.2445834
90	C	9.67645885	3.3420356	12.6098031
91	C	11.9880355	4.23107445	12.8662798
92	C	10.8999161	3.72544861	14.7756247
93	C	12.1345303	4.13945448	15.1224199
94	C	14.2014484	4.78039313	13.8490914
95	C	14.7687219	7.04446337	12.9959239
96	C	14.772173	6.78708075	15.2441998
97	C	15.1939204	8.03824291	14.9754417
98	C	15.6768041	9.36087029	12.8907047

99	C	14.6095481	10.3500502	12.5343358
100	C	13.8315896	11.1620023	13.3539215
101	C	13.0805477	11.9297558	12.4680787
102	C	12.1000563	13.0295431	12.74008
103	H	11.4216094	13.0020299	15.4559819
104	H	8.84067602	12.1604176	15.9122746
105	H	7.21255268	11.8999005	12.5138701
106	H	6.86461968	12.1545865	14.2315701
107	H	6.78209636	10.098271	15.8031615
108	H	5.98237007	7.54152211	15.1697519
109	H	5.29196684	6.5385948	12.9815033
110	H	5.68548233	7.34955951	11.4703519
111	H	7.78283397	5.15360588	14.1283274
112	H	9.16389227	2.57561795	13.1959931
113	H	10.0518621	2.86164658	11.7033509
114	H	10.0837675	3.36248083	15.3816715
115	H	12.6069399	4.20498521	16.0907372
116	H	14.5611134	4.46937134	12.8688273
117	H	14.7387703	4.21429796	14.6122189
118	H	14.6706977	6.26737537	16.1847982
119	H	15.5294729	8.82415338	15.6348948
120	H	16.1834666	9.02328967	11.9836954
121	H	16.4312025	9.82679739	13.5292217
122	H	13.8375469	11.2132046	14.4335177
123	H	12.5490362	13.7954523	13.3768829
124	H	11.8165624	13.5119084	11.8018203
125	C	10.4763997	7.08448092	9.04711323
126	C	11.1580486	7.47509736	7.73105304
127	C	11.2445734	8.99850767	7.82979349
128	C	11.6388586	9.21865035	9.29291618
129	C	10.9209685	8.12032213	10.0977001
130	H	12.1663854	7.05074401	7.68400256
131	H	10.6107765	7.12636655	6.84741964
132	H	10.2625522	9.43994322	7.6271741
133	H	11.9590567	9.43595568	7.12311621
134	H	11.3921525	10.2185245	9.65885547
135	H	12.7223589	9.08503486	9.39314581
136	H	10.0570787	8.5193278	10.6431544
137	H	11.5867095	7.68090064	10.8502554
138	H	10.7228808	6.06680549	9.35593146
139	H	9.39045336	7.14222653	8.91534989

11. References

[1] P. J. Altmann, C. Jandl, A. Pöthig, "Introducing a pyrazole/imidazole based hybrid cyclophane: a hydrogen bond sensor and binucleating ligand precursor" *Dalton Trans.* **2015**, *44*, 11278.

[2] P. J. Altmann, A. Pöthig, "A pH-Dependent, Mechanically Interlocked Switch: Organometallic [2]Rotaxane vs. Organic [3]Rotaxane" *Angew. Chem. Int. Ed.* **2017**, *56*, 15733.

[3] M. Loos, C. Gerber, F. Corona, J. Hollender, H. Singer, "Accelerated isotope fine structure calculation using pruned transition trees" *Anal. Chem.* **2015**, *87*, 5738.

[4] S. Creager, in *Handbook of Electrochemistry*, Elsevier, **2007**, pp. 57.

[5] M. E. Tessensohn, S. J. Ng, K. K. Chan, S. L. Gan, N. F. Sims, Y. R. Koh, R. D. Webster, "Impurities in nitrile solvents commonly used for electrochemistry, and their effects on voltammetric data" *ChemElectroChem* **2016**, *3*, 1753.

[6] F. Neese, Diploma thesis, University of Konstanz (Germany), **1993**.

[7] D. O. Soloviev, C. A. Hunter, "Musketeer: a software tool for the analysis of titration data" *Chem. Sci.* **2024**, *15*, 15299.

[8] Bruker, APEX 4 (2022.10-0), Suite of Crystallographic Software, Madison, Wisconsin (USA), **2022**.

[9] Bruker, SAINT (8.40A), Madison, Wisconsin (USA), **2019**.

[10] Bruker, SADABS (2016/2), Madison, Wisconsin (USA), **2016**.

[11] G. M. Sheldrick, "SHELXT - integrated space-group and crystal-structure determination" *Acta Crystallogr., Sect. A: Found. Crystallogr.* **2015**, *71*, 3.

[12] G. M. Sheldrick, "Crystal structure refinement with SHELXL" *Acta Crystallogr., Sect. C: Cryst. Struct. Commun.* **2015**, *71*, 3.

[13] C. B. Hübschle, G. M. Sheldrick, B. Dittrich, "ShelXle: a Qt graphical user interface for SHELXL" *J. Appl. Crystallogr.* **2011**, *44*, 1281.

[14] D. Kratzert, J. J. Holstein, I. Krossing, "DSR: enhanced modelling and refinement of disordered structures with SHELXL" *J. Appl. Crystallogr.* **2015**, *48*, 933.

[15] D. Kratzert, I. Krossing, "Recent improvements in DSR" *J. Appl. Crystallogr.* **2018**, *51*, 928.

[16] A. Spek, "PLATON SQUEEZE: a tool for the calculation of the disordered solvent contribution to the calculated structure factors" *Acta Crystallogr., Sect. C: Cryst. Struct. Commun.* **2015**, *71*, 9.

[17] *International Tables for Crystallography, Volume C: Mathematical, physical and chemical tables*, International Union of Crystallography, Chester, England, **2006**.

[18] C. R. Groom, I. J. Bruno, M. P. Lightfoot, S. C. Ward, "The Cambridge Structural Database" *Acta Crystallogr., Sect. B: Struct. Sci.* **2016**, *72*, 171.

[19] The PyMOL Molecular Graphics System (Version 2.5.2), Schrödinger, LLC.

[20] C. F. Macrae, I. Sovago, S. J. Cottrell, P. T. A. Galek, P. McCabe, E. Pidcock, M. Platings, G. P. Shields, J. S. Stevens, M. Towler, P. A. Wood, "Mercury 4.0: from visualization to analysis, design and prediction" *J. Appl. Crystallogr.* **2020**, *53*, 226.

[21] W. M. Haynes, *CRC Handbook of Chemistry and Physics*, 96th ed., CRC Press, **2015**.

[22] F. Neese, "The ORCA program system" *Wiley Interdiscip. Rev.: Comput. Mol. Sci.* **2012**, *2*, 73.

[23] F. Neese, "Software update: The ORCA program system—Version 5.0" *Wiley Interdiscip. Rev.: Comput. Mol. Sci.* **2022**, *12*, e1606.

[24] N. Mardirossian, M. Head-Gordon, "ωB97X-V: a 10-parameter, range-separated hybrid, generalized gradient approximation density functional with nonlocal correlation, designed by a survival-of-the-fittest strategy" *Phys. Chem. Chem. Phys.* **2014**, *16*, 9904.

[25] O. A. Vydrov, T. Van Voorhis, "Nonlocal van der Waals density functional: the simpler the better" *J. Chem. Phys.* **2010**, *133*, 244103.

[26] F. Weigend, R. Ahlrichs, "Balanced basis sets of split valence, triple zeta valence and quadruple zeta valence quality for H to Rn: Design and assessment of accuracy" *Phys. Chem. Chem. Phys.* **2005**, *7*, 3297.
[27] D. Bykov, T. Petrenko, R. Izsák, S. Kossmann, U. Becker, E. Valeev, F. Neese, "Efficient implementation of the analytic second derivatives of Hartree–Fock and hybrid DFT energies: a detailed analysis of different approximations" *Mol. Phys.* **2015**, *113*, 1961.
[28] F. Neese, "The SHARK integral generation and digestion system" *J. Comput. Chem.* **2023**, *44*, 381.
[29] F. Weigend, "Accurate Coulomb-fitting basis sets for H to Rn" *Phys. Chem. Chem. Phys.* **2006**, *8*, 1057.
[30] F. Neese, "An improvement of the resolution of the identity approximation for the formation of the Coulomb matrix" *J. Comput. Chem.* **2003**, *24*, 1740.
[31] F. Neese, F. Wennmohs, A. Hansen, U. Becker, "Efficient, approximate and parallel Hartree–Fock and hybrid DFT calculations. A 'chain-of-spheres' algorithm for the Hartree–Fock exchange" *Chem. Phys.* **2009**, *356*, 98.
[32] B. Helmich-Paris, B. de Souza, F. Neese, R. Izsák, "An improved chain of spheres for exchange algorithm" *J. Chem. Phys.* **2021**, *155*, 104109.
[33] G. A. Zhurko, Chemcraft - Graphical Program for Visualization of Quantum Chemistry Computations (Version 1.8), Ivanovo (Russia), **2005**.
[34] T. Lu, F. Chen, "Multiwfn: a multifunctional wavefunction analyzer" *J. Comput. Chem.* **2012**, *33*, 580.
[35] T. Lu, "A comprehensive electron wavefunction analysis toolbox for chemists, Multiwfn" *J. Chem. Phys.* **2024**, *161*.
